# Supplementary material for: A robust yeast biocontainment system with two-layered regulation switch dependent on unnatural amino acid
Source: Nat Commun. 2023 Oct 14;14:6487. doi: 10.1038/s41467-023-42358-4 (PMC10576815; doi:10.1038/s41467-023-42358-4)
Supplement: Supplementary file 1 — Supplementary Information [file 41467_2023_42358_MOESM1_ESM.pdf]

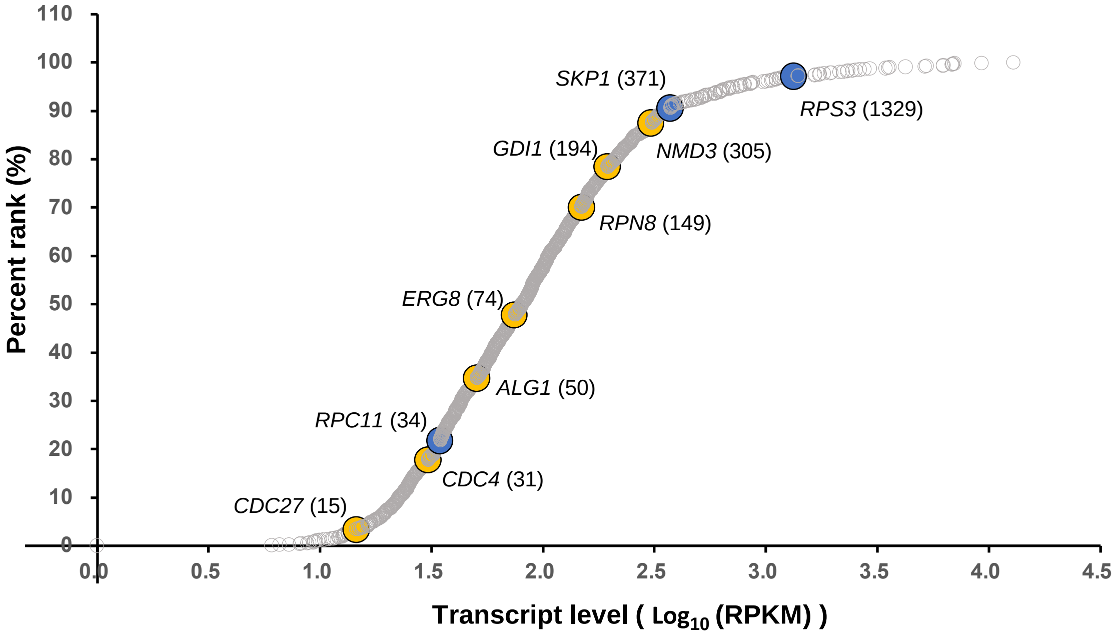

Supplementary Figure 1. Distribution of transcript levels of all essential genes in budding yeast. The percent rank is displayed on the y-axis (the rank of the RPKM value of each essential gene in descending order divided by the total number of essential genes). The transcript level based on Log_{10}(RPKM) value of essential genes is shown on the x-axis. The gray dot represents different essential genes. The essential genes used for OMeY-dependent and transcriptional-based biocontainment strategies are labeled in yellow and blue respectively. The numbers in parentheses indicate the RPKM value of selected genes. Source data are provided as a Source Data file.

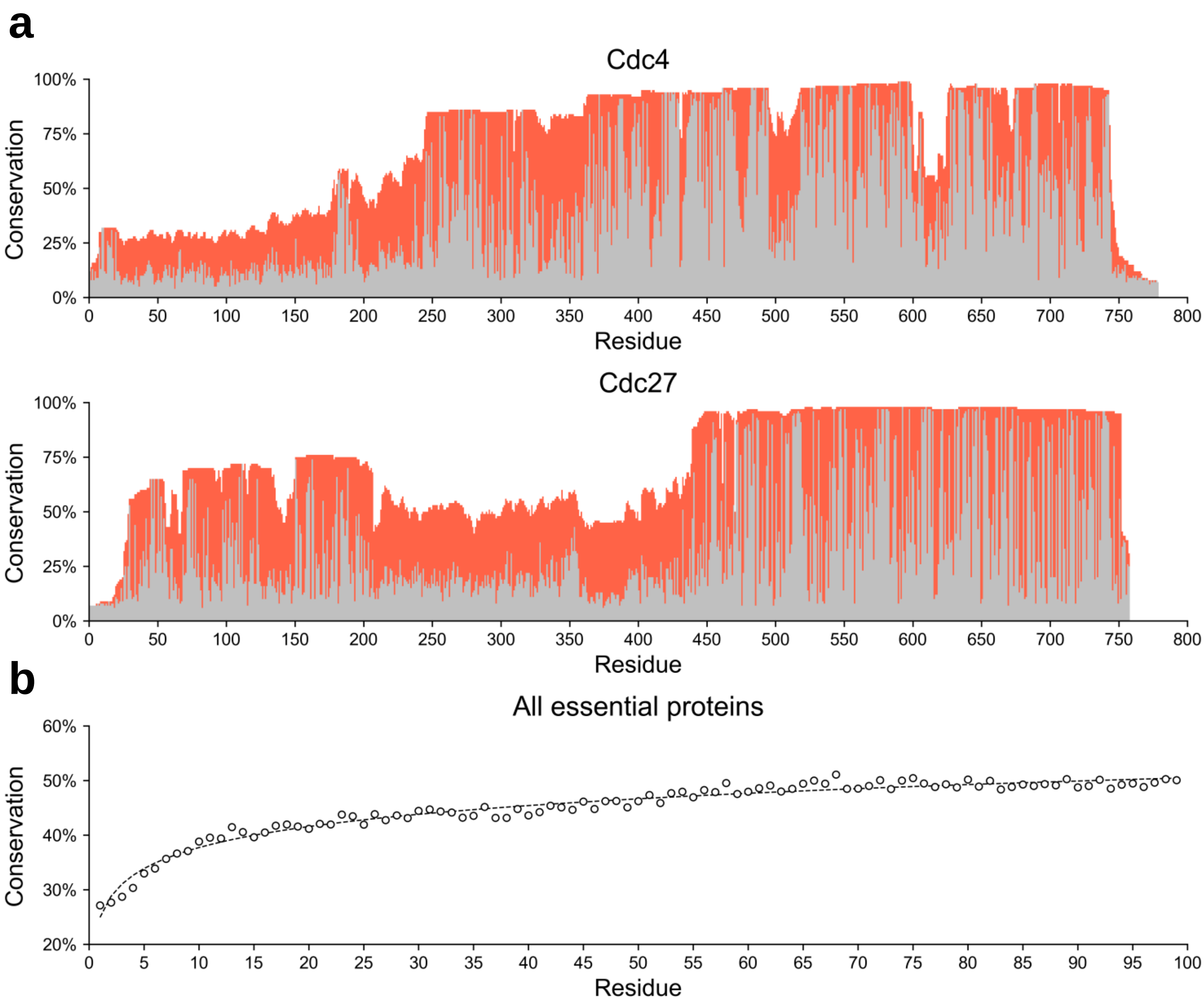

Supplementary Figure 2. Conservation analysis of each residue of essential proteins in budding yeast. **a** Conservation analysis of Cdc4 and Cdc27 proteins. The conservation (y-axis) is represented by the proportion of identical (gray) and substituted (red) amino acids for each residue based on the alignment of protein homologs. The white area denotes the proportion of missing amino acids at the corresponding position among protein homologs. **b** Conservation analysis of the first 100 amino acids for all essential proteins in budding yeast. The y-axis represents the average value of conservation for all essential proteins at the first 100 residues.

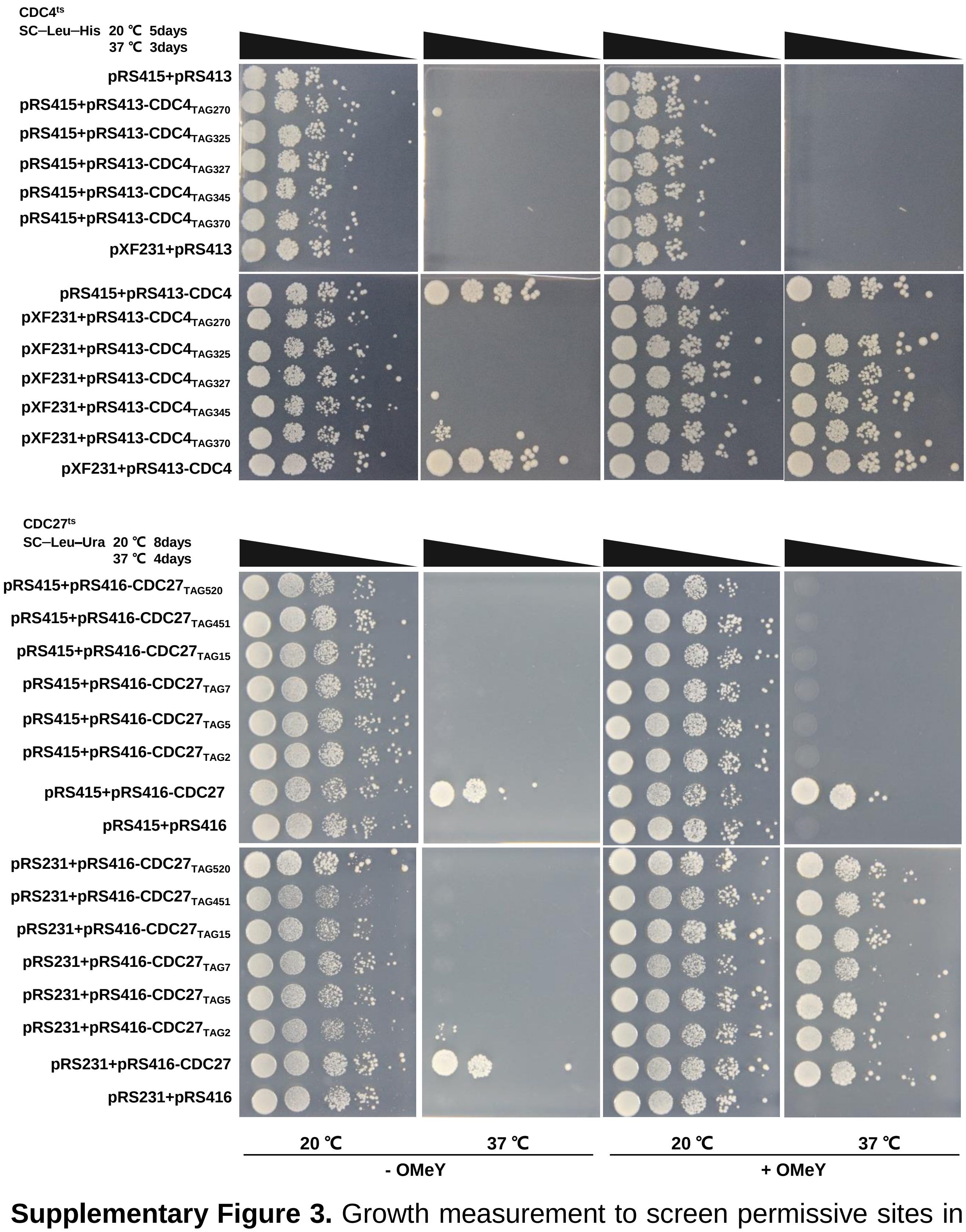

Supplementary Figure 3. Growth measurement to screen permissive sites in Cdc4 and Cdc27 protein for OMeY substitution. The dilution series (by column) of ts mutants co-expressing two plasmids encoding the LeuOmeRS/tRNA$_{CUA}$ pair or empty vector (pRS415) and wild-type *CDC4/27* genes or variants were grown on the corresponding media under different conditions (with and without 1mM OMeY at 20 ℃ and 37 ℃). CDC4^{ts} and CDC27^{ts} mutants were grown on SC–Leu–His and SC–Leu–Ura media plates, respectively. pXF231 is the pRS415 plasmid expressing the LeuOmeRS/tRNA$_{CUA}$ pair.

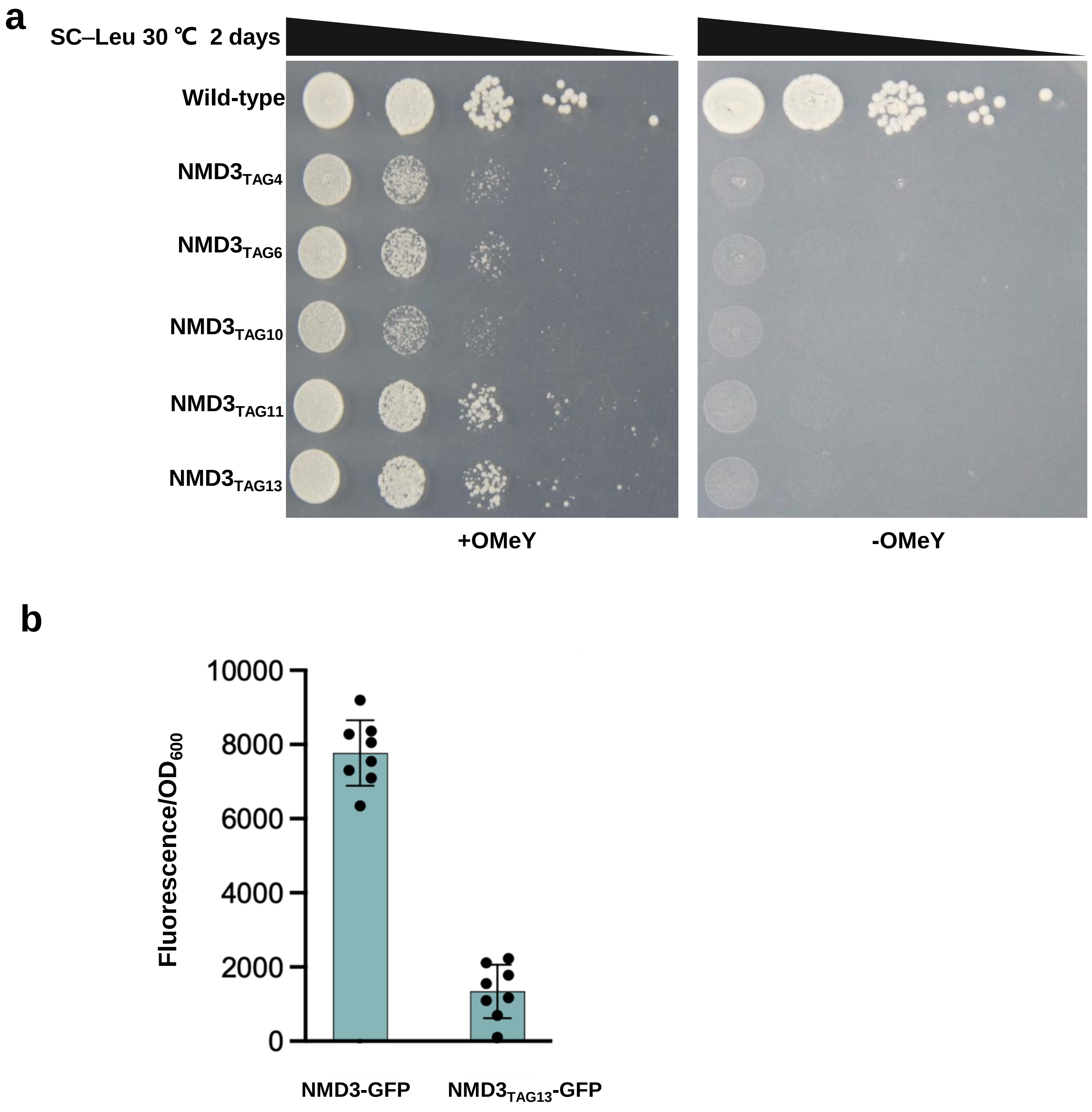

Supplementary Figure 4. Characterization of OMeY autotrophs based on *NMD3* gene. **a** Growth measurement of OMeY-dependent auxotrophs that harbor TAG substitution corresponding to different amino acid residues at the N-terminus of Nmd3 proteins compared with the control strain (BY4741 expressing LeuOmeRS/$tRNA_{CUA}$) . Cells were grown on selective medium plates with and without 1 mM OMeY at 30 ℃ for 2 days. **b.** Fluorescence measurement of strains encoding Nmd3–GFP fusion proteins (both wild-type and $NMD3_{TAG13}$) grown in SC–Leu–Ura medium containing 1 mM OMeY to evaluate amber suppression efficiency. All the data are represented as the mean and standard deviation (n=3 for panel b, n=8 for panel c). Source data are provided as a Source Data file.

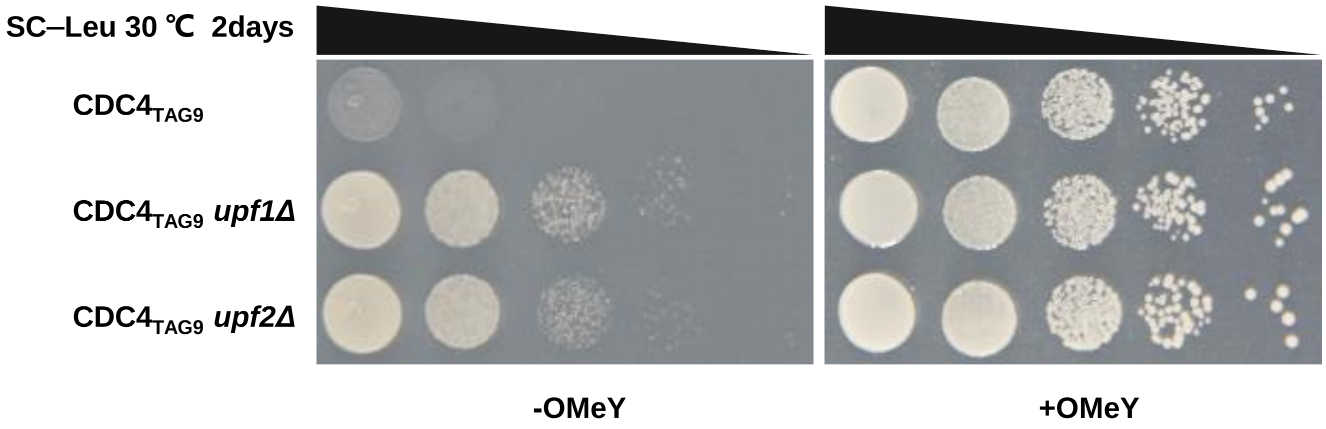

Supplementary Figure 5. Phenotypic assays of mutants with *UPF1* and *UPF2* deletion in comparison with the parental strain (CDC4$_{TAG9}$). Ten-fold serial dilutions of yeast cells were plated on SC–Leu media plates with and without 1 mM OMeY at 30 ℃.

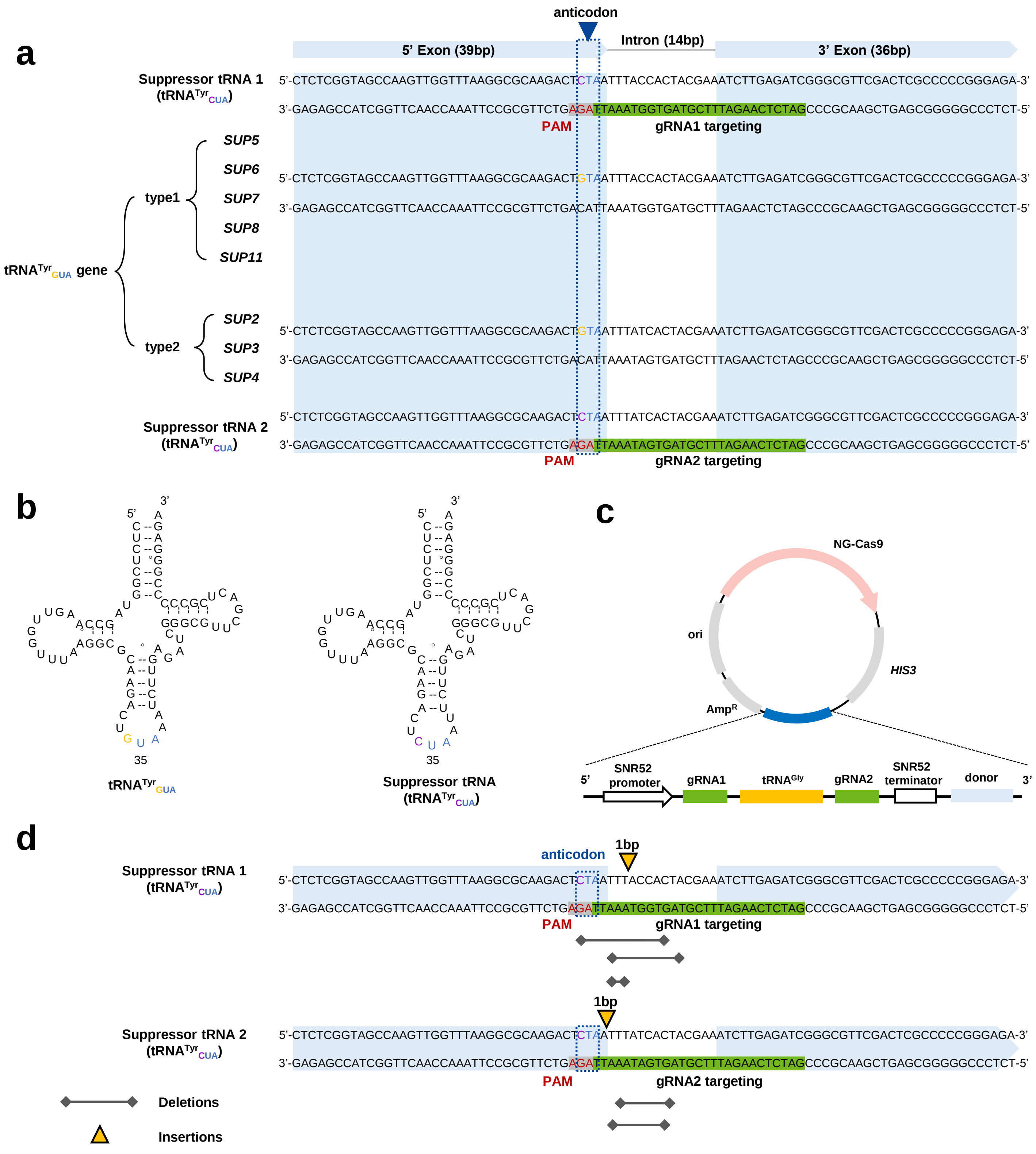

Supplementary Figure 6. Design of CRISPR-Cas9 system for reducing escape frequencies. **a** All tRNA^{Tyr} genes in budding yeast have two types of sequences; the intron sequences differ. SpCas9-NG identified 5'-AGA-3' PAM that is only located at amber suppressor tRNA^{Tyr}. PAM and gRNA target sequences are colored in red and green respectively. The tRNA^{Tyr} exon is shown in the blue region. **b**. Cloverleaf structures of yeast wild-type tRNA$^{Tyr}_{GUA}$ and amber suppressor tRNA$^{Tyr}_{CUA}$. The sequences of wild-type and amber suppressor tRNA^{Tyr} differ at nucleotide 34. The colored nucleotides represent anticodons. Open circles denote non-Watson-Crick base pairs and lines denote base pairs. **c** Schematic of CRISPR-Cas9 system components on plasmid. A tRNA^{Gly} (yellow) is introduced between the gRNA1-gRNA2 array (green) to generate functional gRNAs for editing. The tRNA^{Tyr} gene serving as the donor DNA for the homology-directed repair is labeled in light blue **d** Schematic of the amber suppressor tRNA^{Tyr} targeted for leftover scar interrupts with CRISPR-Cas9 system shown. The yellow triangle indicates corresponding insertion site, and deletions sequence is represented by the location and length of the dark line.

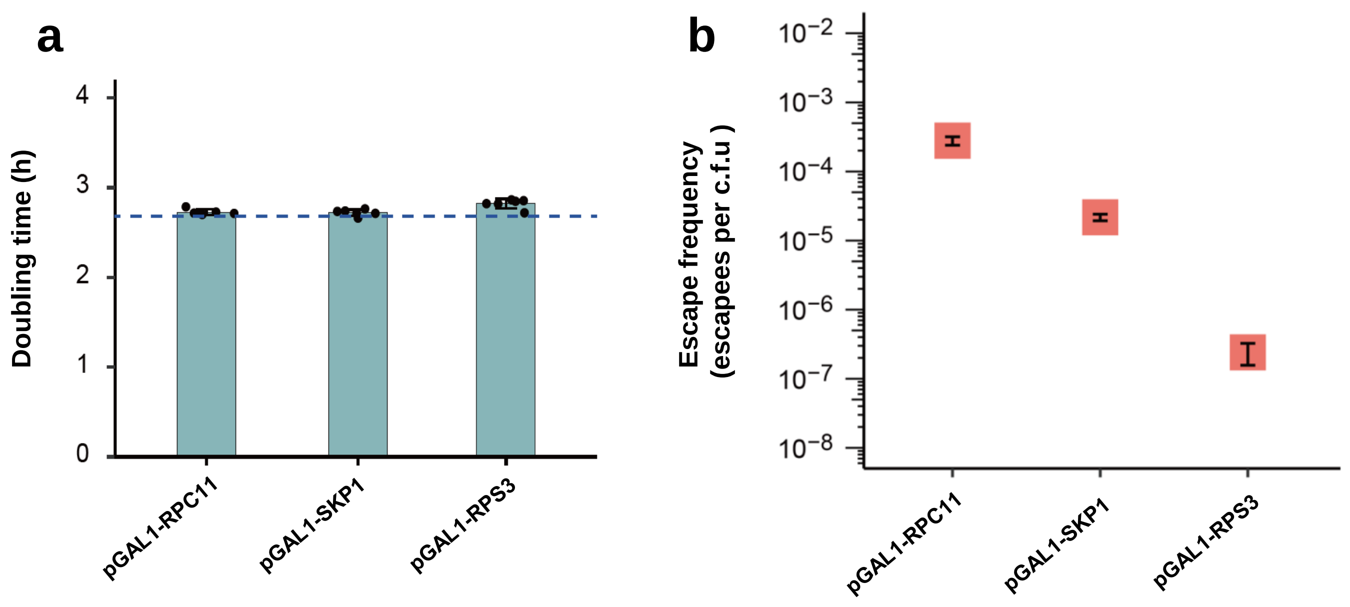

Supplementary Figure 7. Doubling time and escape frequency measurement of the transcriptional-based safeguard strains. **a** Doubling time of all three strains was measured compared with parental strain (BY4741 express LeuOmeRS/$tRNA_{CUA}$) in SC–Leu medium containing 2% (wt/vol) galactose, and the parental strain doubling times showed by the blue dashed horizontal lines. All the data are represented as the mean ± SD (standard deviation) from six biological replicates. **b** Escape frequencies of different transcriptional-based safeguard strains on the day 8. All the data are represented as the mean ±SEM (standard error of the mean) from three biological replicates. Source data are provided as a Source Data file.

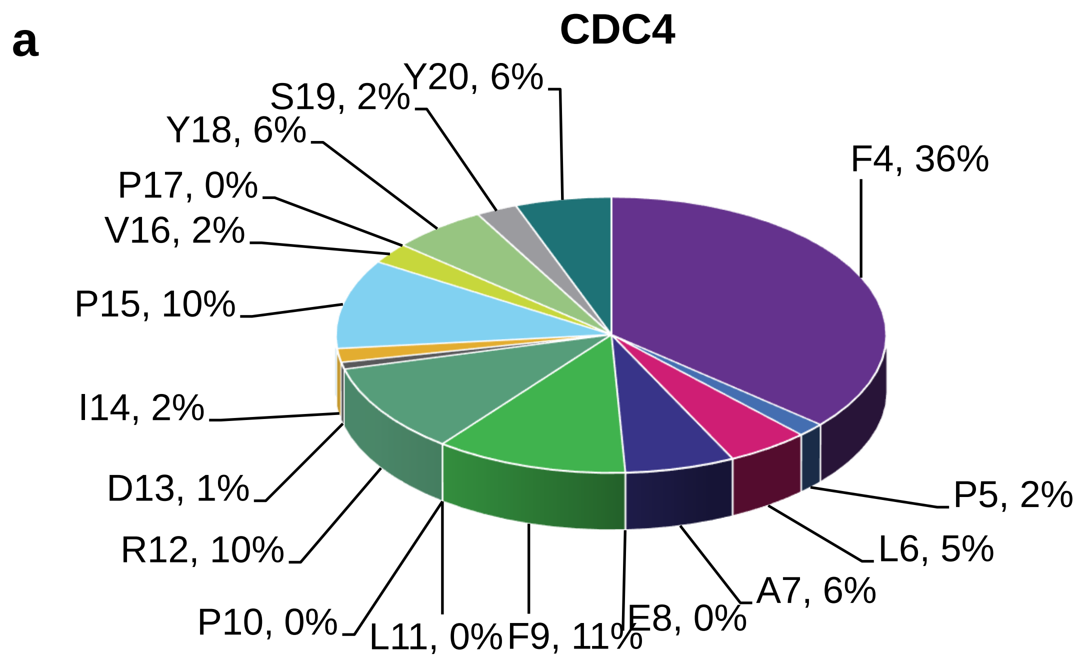

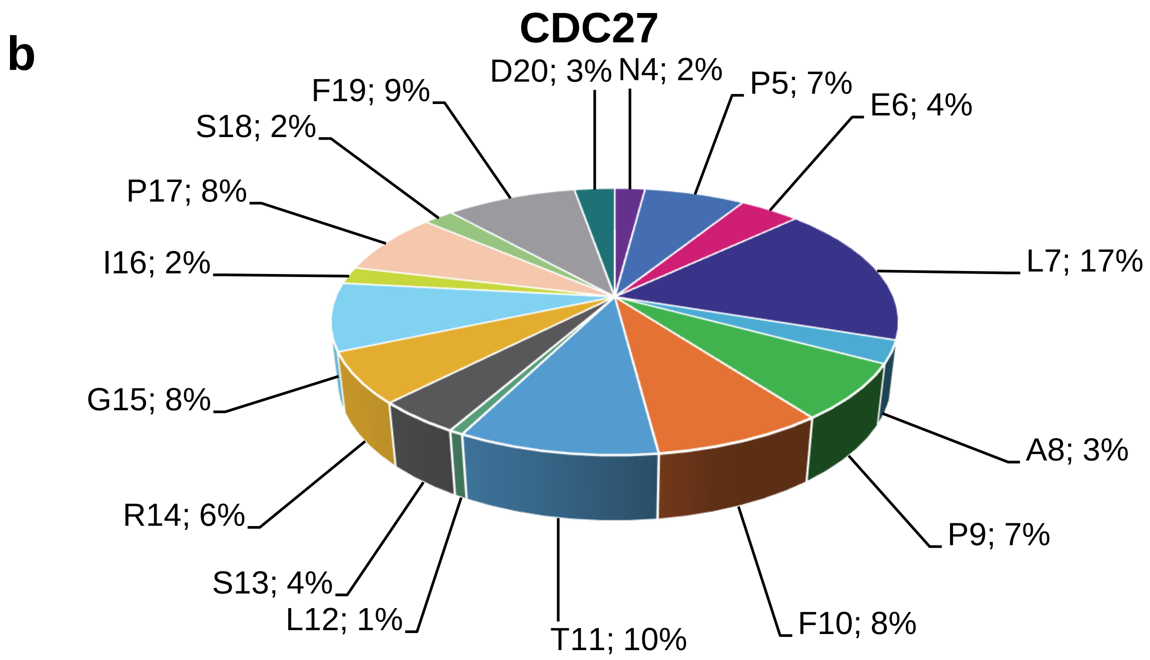

Supplementary Figure 8. Pie charts showing the percentage of TAG substitution corresponding to individual amino acid residues at the N-terminus (from residue 4 to 20) of CDC4 and CDC27. **a.** Total 124 colonies were selected for sanger sequencing of the CDC4 N-terminus. The proportion of TAG codon substitution for each residue is indicated and TAG substitution for residue E8, L11, P10, and P17 was not found. **b.** Total 146 colonies were selected for sanger sequencing of the CDC27 N-terminus. TAG substitution was detected for all tested residues and the proportion of TAG codon substitution for each residue is indicated. Source data are provided as a Source Data file.

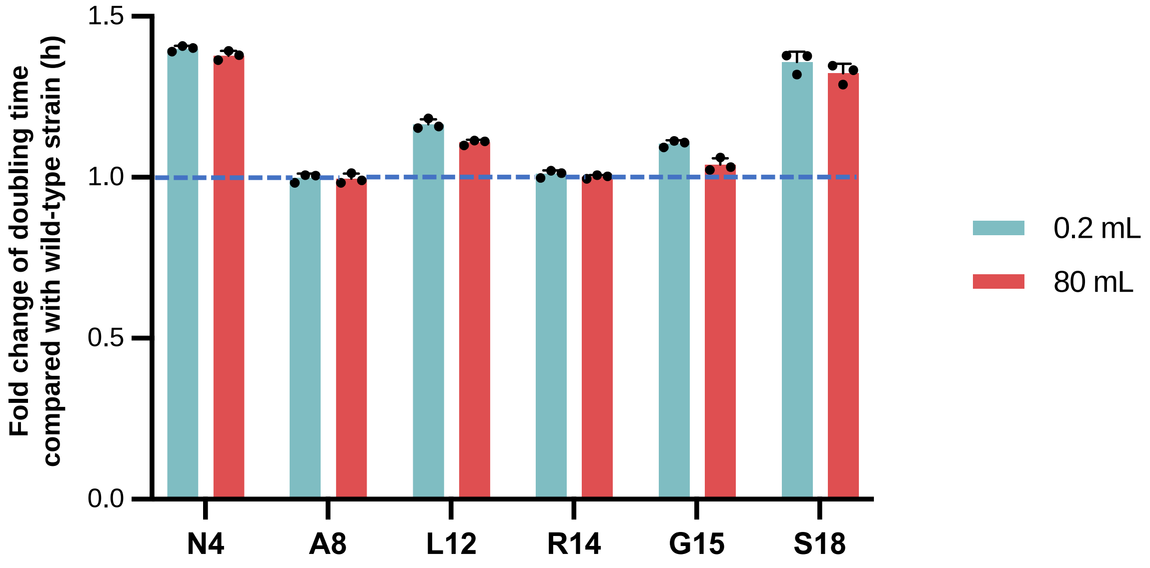

Supplementary Figure 9. Doubling time comparison of different Cdc27-based two-layered safeguard strains grown in small and large scale. Strains were grown in 0.2 mL (blue bars) and 80 mL (red bars) SC–Leu medium containing 1 mM OMeY and 2% (wt/vol) galactose respectively. Fold change of doubling time is shown relative to the growth of pGAL1-RPS3 control strain that contain the *URA3* marker integrated upstream of the WT *CDC27* gene (represented by the blue dashed horizontal line). The x-axis shows different residues of Cdc27 that are replaced by OMeY. The error bars show the mean and standard deviation of three biological replicates. Source data are provided as a Source Data file.

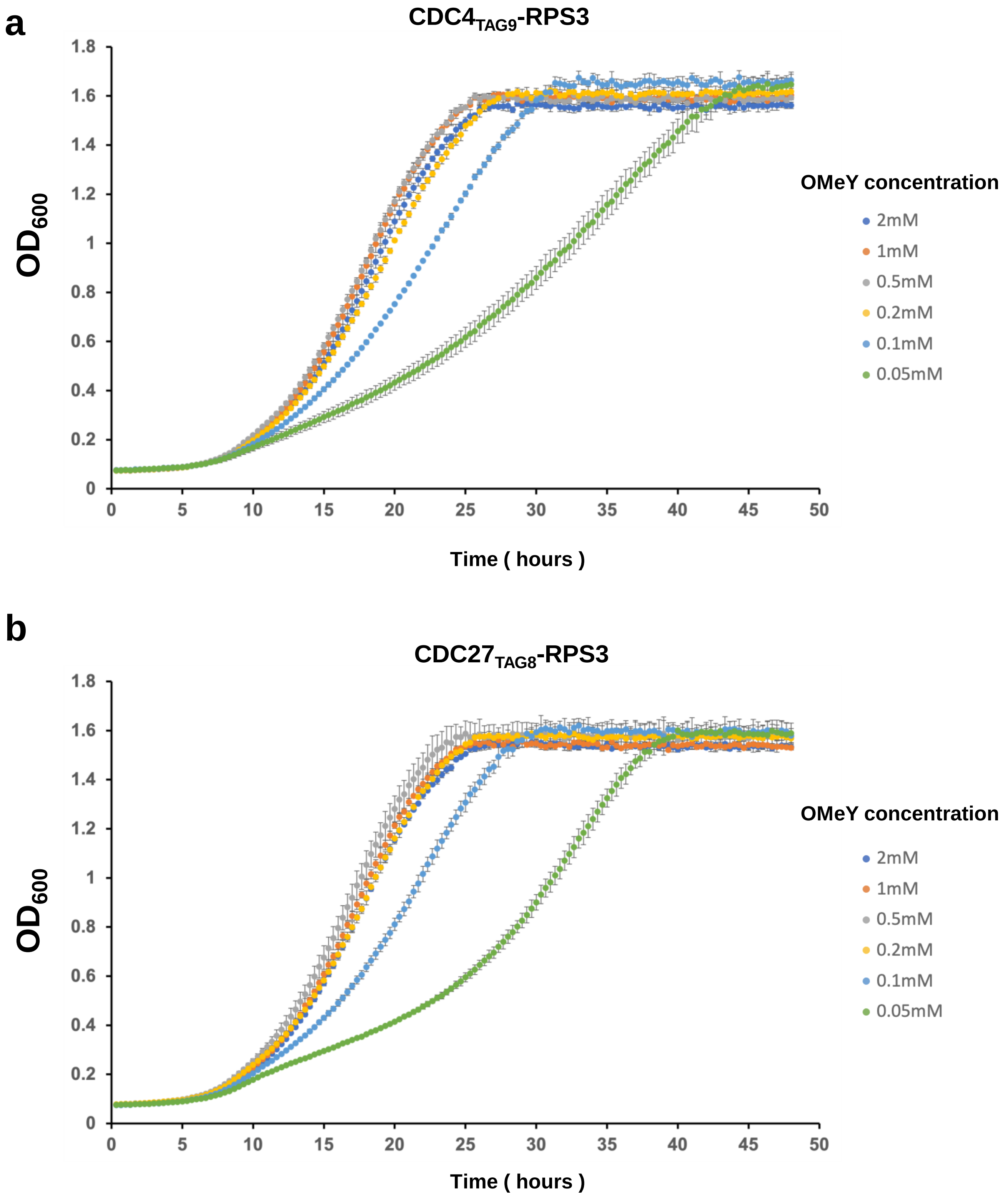

Supplementary Figure 10. The growth curve of multiplex safeguard strains ($CDC4_{TAG9}$-RPS3 and $CDC27_{TAG8}$-RPS3) growing in SC–Leu medium broth containing 2% (wt/vol) galactose and supplemented with different concentration of OMeY (2, 1, 0.5, 0.2, 0.1 and 0.05 mM). Optical densities (OD_{600}) were measured at every 20 minutes. All the data are represented as the mean ± SD (standard deviation) from three biological replicates. Source data are provided as a Source Data file.

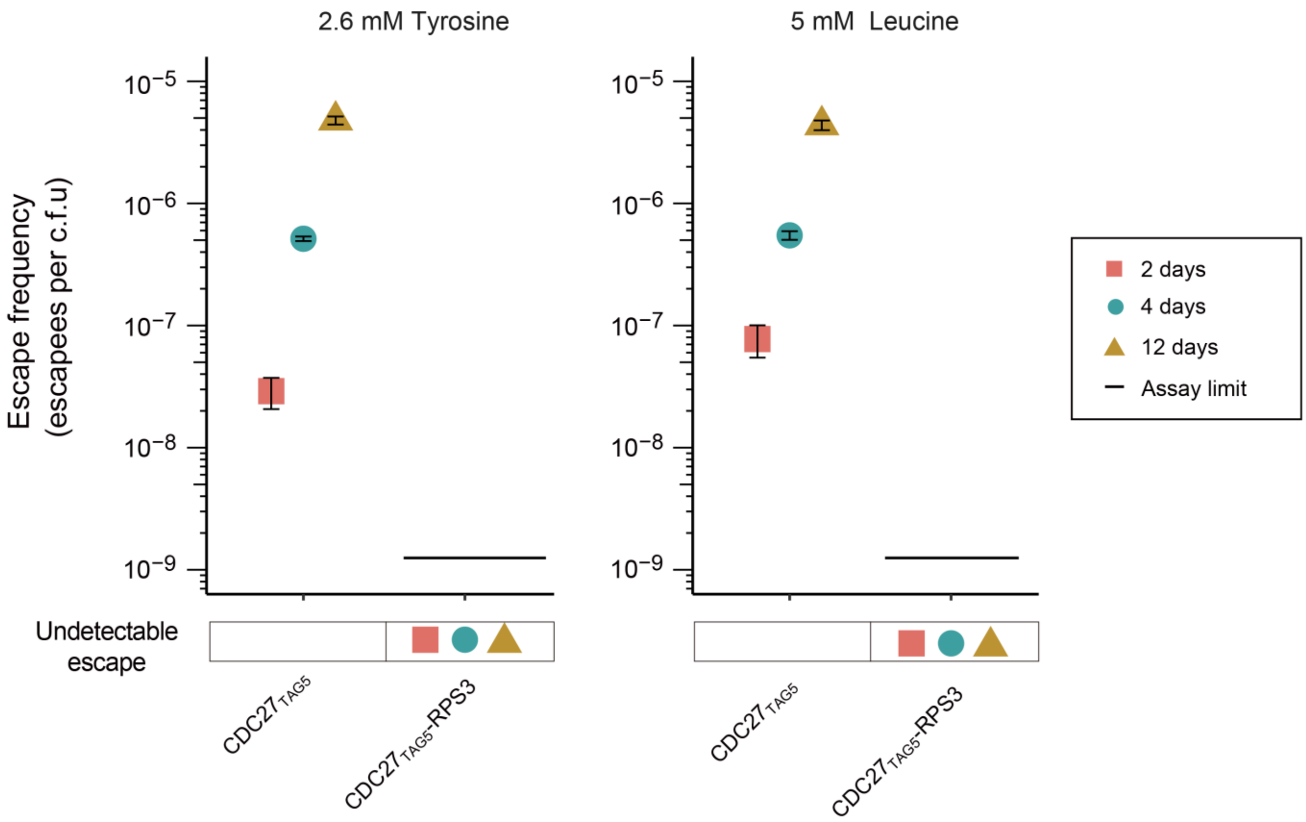

Supplementary Figure 11. Escape frequencies of the OMeY-dependent auxotroph ($CDC27_{TAG5}$) and the two-layered safeguard strain ($CDC27_{TAG5}$-RPS3) in the presence of 5 mM leucine or 2.6 mM tyrosine. Escape frequencies were measured at day 2, 4 and 12 indicated as red square, blue circle and yellow triangle respectively. The assay limit is determined by 1/(total CFU plated) with the assay limit of 1.3×10^{-9} escapees per CFU (see source data for details) and the black line represents undetectable growth. Error bars show the mean ± SEM of six samples including three biological replicates that was conducted in duplicate. Source data are provided as a Source Data file.

Supplementary Tables

Table S1. The reasons of escaper were analyzed by WGS.

Escape mechanisms	OMeY-dependent strain (parental)	Escaper	Gene	Mutation
Mutation of TAG to sense codon	$CDC4_{TAG325}$	yXF260	*CDC4*	OMeY325Q
Occurrence of amber suppressor tRNA	$CDC4_{TAG325}$	yXF258	*SUP7*($tRNA^{Tyr}_{GUA}$)	G34>C in anticodon
	$CDC4_{TAG325}$	yXF259	$tRNA^{Leu}_{CAA}$[a]	A35>U in anticodon
	$CDC4_{TAG325}$	yXF261	*SUP8*($tRNA^{Tyr}_{GUA}$)	G34>C in anticodon
	$CDC27_{TAG5}$	yXF262	*SUP54*($tRNA^{Leu}_{CAA}$)	A35>U in anticodon
	$CDC27_{TAG5}$	yXF263	$tRNA^{Leu}_{CAA}$[b]	A35>U in anticodon
	$CDC27_{TAG5}$	yXF264	*SUP3*($tRNA^{Tyr}_{GUA}$)	G34>C in anticodon
	$CDC27_{TAG520}$	yXF267	*SUP7*($tRNA^{Tyr}_{GUA}$)	G34>C in anticodon
	$CDC27_{TAG520}$	yXF268	*SUP4*($tRNA^{Tyr}_{GUA}$)	G34>C in anticodon
	$CDC27_{TAG520}$	yXF269	*SUP8*($tRNA^{Tyr}_{GUA}$)	G34>C in anticodon
	$CDC27_{TAG520}$	yXF270	*SUP3*($tRNA^{Tyr}_{GUA}$)	G34>C in anticodon
	$CDC27_{TAG520}$	yXF271	*SUP8*($tRNA^{Tyr}_{GUA}$)	G34>C in anticodon
	$CDC27_{TAG520}$	yXF272	*SUP4*($tRNA^{Tyr}_{GUA}$)	G34>C in anticodon
	$CDC27_{TAG451}$	yXF265	$tRNA^{Trp}_{CCA}$[c]	C35>U in anticodon
	$CDC27_{TAG451}$	yXF266	$tRNA^{Trp}_{CCA}$[d]	C35>U in anticodon
Disruption of NMD pathway	$CDC4_{TAG9}$	yXF255	*NAM7*	2670delC (frameshift)
	$CDC4_{TAG9}$	yXF256	*NMD2*	828delC (frameshift)
	$CDC4_{TAG9}$	yXF257	*NAM7*	Y79 (TAA mutation）

tRNA systematic name: [a]YNCL0034W; [b]YNCG0040C; [c]YNCM0022C; [d]YNCG0042C.

Table S2. The leftover scar interrupts analysis based on CRISPR-Cas9 system.

Escaper	OMeY-dependent strain (parental)	Causative interrupts	The leftover scar interrupts location
yXF273	yXF286	*SUP5* amber suppressor	Insertion of 1-bp T at base pair 43
yXF274	yXF286	*SUP3* amber suppressor	8-bp deletion between 41 and 48bp
yXF275	yXF286	*SUP5* amber suppressor	12-bp deletion between 36 and 47bp
yXF276	yXF237	*SUP8* amber suppressor	10-bp deletion between 40 and 49bp
yXF277	yXF237	*SUP4* amber suppressor	8-bp deletion between 40 and 47bp
yXF278	yXF237	*SUP8* amber suppressor	3-bp deletion between 40 and 42bp
yXF279	yXF237	*SUP5* amber suppressor	Insertion of 1-bp T at base pair 43
yXF280	yXF237	*SUP4* amber suppressor	Insertion of 1-bp A at base pair 40

Table S3. List of plasmids used in this study.

plasmid	Description	Source or reference
pXF231	*LEU2,* pRS315 carrying *LeuOmeRS/tRNA*$_{CUA}$	[1]
pXF473	*URA3*, pRS416 carrying *CDC27*	This study
pXF474	*URA3*, pRS416 carrying *CDC27*$_{TAG2}$	This study
pXF475	*URA3*, pRS416 carrying *CDC27*$_{TAG5}$	This study
pXF476	*URA3*, pRS416 carrying *CDC27*$_{TAG7}$	This study
pXF478	*URA3*, pRS416 carrying *CDC27*$_{TAG15}$	This study
pXF480	*URA3*, pRS416 carrying *CDC27*$_{TAG451}$	This study
pXF482	*URA3*, pRS416 carrying *CDC27*$_{TAG520}$	This study
pXF484	*HIS3*, pRS413 carrying *CDC4*	This study
pXF485	*HIS3*, pRS413 carrying *CDC4*$_{TAG270}$	This study
pXF486	*HIS3*, pRS413 carrying *CDC4*$_{TAG325}$	This study
pXF487	*HIS3*, pRS413 carrying *CDC4*$_{TAG327}$	This study
pXF488	*HIS3*, pRS413 carrying *CDC4*$_{TAG345}$	This study
pXF489	*HIS3*, pRS413 carrying *CDC4*$_{TAG370}$	This study
pXF495	*URA3*, pRS416 carrying *NMD3*	This study
pXF496	*URA3*, pRS416 carrying *NMD3*$_{TAG3}$	This study
pXF497	*URA3*, pRS416 carrying *NMD3*$_{TAG364}$	This study
pXF469	*HIS3*, pRS423 carrying P$_{GAL1}$-*SpCas9-NG*-T$_{ADH1}$,P$_{SNR52}$-*gRNA1-SUF17-gRNA2*-T$_{SNR52}$ -*sup8*	This study
pXF500	*HIS3*, pRS423 carrying P$_{GAL1}$- *SpCas9-NG*	This study
pXF509	*HIS3,* pRS313 carrying *LeuOmeRS/tRNA*$_{CUA}$	This study

Table S4. List of primers used in this study.

Primer Pair	Primer Sequence (5'-3')[a]	Description; construct generated
CDC27^{ts}-F	5'- CAAGACATGCTATAGAAAGGCGCTAGCTTGTGATCCTCAGCATTACAATGCATATTACG**ac**ttgggtacgagcgctatgaaattaggtc -3'	Site-directed mutagenesis G613 of Cdc27 to D; yXF243
CDC27^{ts}-R	5'- CGTCAAGACTGTCAAGGAGGGTATTCTGGGCCTCCATGTCatcgcaggaaccgcagacatgatg -3'	
CDC27^{ts}-KanMX-F	5'- gacatggaggcccagaataccctc -3'	The *KanMX* cassette with homologous arm amplification primer;yXF243
CDC27^{ts}-KanMX-R	5'- AAATAAAAAGTTTACGAATTAATACAATCAAATTAGACACTGATACTATCAAGAAGTACcagtatagcgaccagcattcacatacgattg -3'	
CDC27-F	5'- gttggtcccatttggtacgtcttg -3'	*CDC27* amplification primer;pXF473 and yXF216
CDC27-R	5'- atcgcaggaaccgcagacatg -3'	
CDC4-F	5'- gcacttgaggaggattacttttactttgagcc -3'	*CDC4* amplification primer;pXF484 and yXF217
CDC4-R	5'- ccttctcaactaaaaacttgtactttccatattttcaggg -3'	
NMD3-F	5'- gaagtatgtttctatataacgggtgagaaaatcgc -3	*NMD3* amplification primer;pXF495
NMD3-R	5'- gcggaagtttaaaaatcttgcgtaagagctc -3	
CDC27-A2-F	5'- cgaacttttcgtgcaggatacataataaaaatagcatg**ta**ggtaaatcctgagttagcaccgttcaccctctc -3'	Site-directed mutagenesis A2 of Cdc27 to TAG;pXF474
CDC27-A2-R	5'- gagagggtgaacggtgctaactcaggatttacc**ta**catgctatttttattatgtatcctgcacgaaaagttc -3'	
CDC27-P5-F	5'- gtgcaggatacataataaaaatagcatggcggtaaat**tag**gagttagcaccgttcaccctctcgagag -3'	Site-directed mutagenesis P5 of Cdc27 to TAG;pXF475 and yXF218
CDC27-P5-R	5'- ctctcgagagggtgaacggtgctaactc**cta**atttaccgccatgctatttttattatgtatcctgcac -3'	
CDC27-L7-F	5'- ggatacataataaaaatagcatggcggtaaatcctgagt**ag**gcaccgttcaccctctcgag -3'	Site-directed mutagenesis L7 of Cdc27 to TAG;pXF476
CDC27-L7-R	5'- ctcgagagggtgaacggtgc**ct**actcaggatttaccgccatgctatttttattatgtatcctgc -3'	
CDC27-G15-F	5'- gagttagcaccgttcaccctctcgaga**tag**atccccagctttgatgatcaagctttgagc -3'	Site-directed mutagenesis G15 of Cdc27 to TAG;pXF478 and yXF219
CDC27-G15-R	5'- gctcaaagcttgatcatcaaagctggggat**cta**tctcgagagggtgaacggtgctaactc -3'	
CDC27-N451-F	5'- taatttcgctttaatattaaggtcgtcatcacaatac**tag**tcgttcaaggcaataagactgttcgag -3'	Site-directed mutagenesis N451 of Cdc27 to TAG;pXF480 and yXF220
CDC27-N451-R	5'- **cta**gtattgtgatgacgaccttaatattaaagcgaaattatacatg -3'	
CDC27-H520-F	5'- tctactttgctgtggcatttg**tag**gacaaggttaaatcttcaaatttggcaaatgg -3'	Site-directed mutagenesis H520 of Cdc27 to TAG;pXF482 and yXF221
CDC27-H520-R	5'- aagatttaaccttgtc**cta**caaatgccacagcaaagtagaaaaaatttccatatc -3'	
CDC27-URA3-F	5'- gagagtgcaccataccacagcttttc -3'	The *URA3* cassette with homologous arm about *CDC27* amplification primer;yXF218,yXF219, yXF220 and yXF221
CDC27-URA3-R	5'- AAATAAAAAGTTTACGAATTAATACAATCAAAT	

	TAGACACTGATACTATCAAGAAGTACcggtattttctccttacgcatctgtgc -3'	
CDC27-BleoR-F	5'- gacatggaggcccagaataccctc -3'	The *BleoR* cassette with homologous arm about *CDC27* amplification primer;yXF230
CDC27-BleoR-R	5'- TAATACAATCAAATTAGACACTGATACTATCAAGAAGTACccggtagaggtgtggtcaataagagc -3'	
CDC4-F9-F	5'- tgagt**ag**ccattacgtgatatccctgttccttatagctac	Site-directed mutagenesis F9 of Cdc4 to TAG;yXF222
CDC4-F9-R	5'- gtagctataaggaacagggatatcacgtaatgg**ct**actcagctaagggaaacgaccccatc -3'	
CDC4-L270-F	5'- actttaatcaaggataatt**ag**aagagggacctaataacgtctttgcctttg -3'	Site-directed mutagenesis L270 of Cdc4 to TAG;pXF485
CDC4-L270-R	5'- acgttattaggtccctctt**ct**aattatccttgattaaagtccccaagtcggatagttc -3'	
CDC4-F325-F	5'- ctgatatcggaaaatt**ag**gtgagcccaaagggttttaattctctcaatc -3'	Site-directed mutagenesis F325 of Cdc4 to TAG;pXF486 and yXF223
CDC4-F325-R	5'- aattaaaaccctttgggctcac**ct**aattttccgatatcagaagtttttccacaacgatgtag -3'	
CDC4-S327-F	5'- cttctgatatcggaaaattttgtg**tag**ccaaagggttttaattctctcaatctcaaactctcc -3'	Site-directed mutagenesis S327 of Cdc4 to TAG;pXF487 and yXF224
CDC4-S327-R	5'- ttgg**cta**cacaaaattttccgatatcagaagtttttccacaacg -3'	
CDC4-L345-F	5'- aatacccaaaa**tag**tcacaacaagatcgccttagattatcttttctgg -3'	Site-directed mutagenesis L345 of Cdc4 to TAG;pXF488 and yXF225
CDC4-L345-R	5'- gataatctaaggcgatcttgttgtga**cta**ttttgggtatttttgggagagtttgagattgag -3'	
CDC4-F370-F	5'- cattttaaaaaattggtacaatcccaagt**ag**gtaccacaaaggaccacgttaagaggc -3'	Site-directed mutagenesis F370 of Cdc4 to TAG;pXF489
CDC4-F370-R	5'- ttgtggtac**ct**acttgggattgtaccaattttttaaaatgaatatattctcc -3'	
CDC4-URA3-F	5'- gagagtgcaccataccacagcttttc -3'	The *URA3* cassette with homologous arm about *CDC4* amplification primer;yXF222, yXF223, yXF224 and yXF225
CDC4-URA3-R	5'- CTTTTATATTTTTTTAGATCATTTTCAAAACTTTCTTAAACcggtattttctccttacgcatctgtgc -3'	
NMD3-F3-F	5'- aatttgacaaaaggatggaat**ag**acacctatagatccgcaccagc -3'	Site-directed mutagenesis F3 of Nmd3 to TAG;pXF496
NMD3-F3-R	5'- tgcggatctataggtgt**ct**attccatccttttgtcaaattcctcaacg -3'	
NMD3-F364-F	5'- atctcggtggtatttgc**tag**gcaggtgatagtgttatgggttatttcattgc -3'	Site-directed mutagenesis F364 of Nmd3 to TAG;pXF497
NMD3-F364-R	5'- cccataacactatcacctgc**cta**gcaaataccaccgagatgagacctg -3'	
ALG1-F250-F	5'- **tag**ataaagaattatattcgcgatgattttgatacagaaaaaggc -3'	Site-directed mutagenesis F250 of Alg to TAG;yXF226
ALG1-F250-R	5'- tttctgtatcaaaatcatcgcgaatataattctttat**ct**aggctttggtagttagggctttttgacg -3'	

ALG1-URA3-F	5'- gagagtgcaccataccacagcttttc -3'	The *URA3* cassette with homologous arm about *ALG1* amplification primer;yXF226
ALG1-URA3-R	5'- GCGCCATCTTCTAACGCCGGCTATGATAACATTTTAGGAAcggtattttctccttacgcatctgtgc -3'	
ERG8-Y401-F	5'- aggagttcttacttgcttaatacctggtgctggtggtta**g**gacgccattgcagtgattactaagc -3'	Site-directed mutagenesis Y401 of Erg8 to TAG;yXF227
ERG8-Y401-R	5'- **c**taaccaccagcaccaggtattaagcaag -3'	
ERG8-URA3-F	5'- gagagtgcaccataccacagcttttc -3'	The *URA3* cassette with homologous arm about *ERG8* amplification primer;yXF227
ERG8-URA3-R	5'- TAAATGACATAAAGTTATATATCTAGAAAGTTTATTTATCcggtattttctccttacgcatctgtgc -3'	
RPN8-K7-F	5'- caaattacaatgtctctacaacacgag**tag**gttaccattgcaccactagttttgctatc -3'	Site-directed mutagenesis K7 of Rpn8 to TAG;yXF228
RPN8-K7-R	5'- caatggtaacc**cta**ctcgtgttgtagagacattgtaatttgattttcc -3'	
RPN8-URA3-F	5'- gagagtgcaccataccacagcttttc -3'	The *URA3* cassette with homologous arm about *RPN8* amplification primer;yXF228
RPN8-URA3-R	5'- ACGAGAATAGAGGCGATATTATGCATTTATATTTGAAATTcggtattttctccttacgcatctgtgc -3'	
GDI1-T5-F	5'- **tag**atagacactgactacgacgtgattgtc -3'	Site-directed mutagenesis T5 of Gdi1 to TAG;yXF229
GDI1-T5-R	5'- cggtacctaagacaatcacgtcgtagtcagtgtctat**cta**ttcttgatccattgtgttatgctatataagtgg -3'	
GDI1-URA3-F	5'- gagagtgcaccataccacagcttttc -3'	The *URA3* cassette with homologous arm about *GDI1* amplification primer;yXF229
GDI1-URA3-R	5'- TTATGAATCAATCAGAATACGCATATAACAAAAAAAAAAAcggtattttctccttacgcatctgtgc -3'	
Pgal1-RPC11-F	5'- Y3GAATGCTGGTCGCTATACTGggatggacgcaaagaagtttaataatcatattac -3'	The P_{gal1} cassette with homologous arm about *RPC11* amplification primer;yXF239
Pgal1-RPC11-R	5'- TCAGTAGCATATTGTTACACGAAGGACAAAACGAAAGCATtatagttttttctccttgacgttaaagtatagagg -3'	
RPC11-KanMX-F	5'- TTTTGTGTTGCTCATGTCATAGCATTCAACTACAGATGAAgacatggaggcccagaataccctc -3'	The *KanMX* cassette with homologous arm about *RPC11* amplification primer;yXF239
RPC11-KanMX-R	5'- AAACTTCTTTGCGTCCATCCcagtatagcgaccagcattcacatacgattg -3'	
Pgal1-SKP1-F	5'- GAATGCTGGTCGCTATACTGggatggacgcaaagaagtttaataatcatattac -3'	The P_{gal1} cassette with homologous arm about *SKP1* amplification primer;yXF240
Pgal1-SKP1-R	5'- GTGAATCGTTCACCCTCACCACTCACTAGGACAACATTAGAAGTCACCATtatagttttttctccttgacgttaaagtatagagg -3'	
SKP1-KanMX-F	5'- GACATTACCCTGCAGCGATTTAACGGGCGTCTTGGAGAAAAGAAGAGAAAgacatggaggcccagaataccctc -3'	The *KanMX* cassette with homologous arm about *SKP1* amplification primer;yXF240
SKP1-KanMX-R	5'- AAACTTCTTTGCGTCCATCCcagtatagcgaccagcattcacatacgattg -3'	
Pgal1-RPS3-F	5'- GAATGCTGGTCGCTATACTGggatggacgcaaagaagtttaataatcatattac -3'	The P_{gal1} cassette with homologous arm about *RPS3* amplification primer;yXF241
Pgal1-RPS3-R	5'- TTCAGCGTAGAAGACACCGTCAGCGACTAGCTTTCTTTTCTTAGAGATTAAAGCGACCATtatagttttttctccttgacgttaaagtatagagg -3'	

RPS3-KanMX-F	5'- GTAAATTCTCTTGTTTTTTTTTCTTGATTTCACTTCTTTTCATGTTCCTTTGGAATAATCgacatggaggcccagaataccctc -3'	The *KanMX* cassette with homologous arm about *RPS3* amplification primer;yXF241
RPS3-KanMX-R	5'- AAACTTCTTTGCGTCCATCCcagtatagcgaccagcattcacatacgattg -3'	
P_{CDC27}-URA3-F	5'- CTCGCCAAAAGCCATCTTCGTAC -3'	The *URA3* cassette with homologous arm about *CDC27* amplification primer;yXF303~319
P_{CDC27}-URA3-R	5'- CTTGATTTATCGAGAATGGAGCATTCAGC -3'	
P_{CDC4}-URA3-F	5'- CCACTGAGAGCTGTTCGTAGATAAATC -3'	The *URA3* cassette with homologous arm about *CDC4* amplification primer;yXF290~302
P_{CDC4}-URA3-R	5'- CTTCTGTCTCAACTGTCTTAGCC -3'	
SpCas9-NG-F	5'- ggattataaagatgacgatgacaaacctcc -3'	*SpCas9-NG* amplification primer; pXF469 and pXF500
SpCas9-NG-R	5'- gctatacctgagaaagcaacctgacctac -3'	
YPL062W-HIS-F	5'- cacatacgacactgcccctcacgtaagggcCACACCGCATAGATCCGTCGAGTTC -3'	Primers amplificate the tGeS cassette;yXF281 and yXF282
YPL062W-HoR-R	5'- ggttcagcagtgtcaaagtg -3'	
KanMX-F	5'-cggtttggttgatgcgagtg -3'	Primer for specific gene *KanMX*
KanMX-R	5'-cgattccgactcgtccaaca -3'	
P_{NMD3}-URA3-F	5'- AAGAGGGAATCGCTGATGGC -3'	The *URA3* cassette with homologous arm about *NMD3* amplification primer;yXF321-325
P_{NMD3}-URA3-R	5'- TCACAGTTTCTGCAAAATGAAATGTTCG -3'	
NMD3-GFP-F	5'- ATCTCAGCAGggatccgctggctccgctgctggttctggcgaagcatccaagggcgaggagc -3'	The *GFP* cassette with homologous arm about *NMD3* and KanMX amplification primer;yXF454 and yXF455
GFP-KanMX-R	5'- ATCTCGAGCTATTATTAGTGgtggtggtg -3'	
GFP-KanMX-F	5'- CACTAATAATAGCTCGAGATctcttaacaaacactagcatttcagtttatc -3'	The *KanMX* cassette with homologous arm about *GFP* and *NMD3* amplification primer; yXF454 and yXF455
KanMX-NMD3-R	5'- TCTTGCGTAAGAGCTCTACAAATGAAAAATCTTGCTTGCCTATCTAGTTTTgacatggaggcccagaataccc -3'	
pXF231-HIS-F	5'- ggagaaaataccgcatcaggaaattgtaaa -3'	The pXF231 amplification primer via inverse PCR; pXF509
pXF231-HIS-R	5'- agtacaatctgctctgatgccgcatagtta -3'	
pXF231-HIS-R	5'- GTTTACAATTTCCTGATGCGGTATTTTCTCCgcattagtcagggaagtcataacacagtcc -3'	Primer to amplify *HIS3* cassette with homologous arm on pXF231; pXF509
pXF231-HIS-F	5'- TAACTATGCGGCATCAGAGCAGATTGTACTcacaccgcatagatccgtcgagttc -3'	

[a] Bolded are the locations for site-directed mutagenesis, while uppercase letters indicate oligonucleotide sequences added to the primers to facilitate cloning.

Table S5. List of strains used in this study.

Strain	Description	Source or reference
**Strains:**		
S. cerevisiae		
yXF215	*MATa ura3Δ0 leu2Δ0 his3Δ1 met15Δ0 [pXF231]*	This study
yXF216	*yXF215 Δcdc27::CDC27-URA3*	This study
yXF218	*yXF215 Δcdc27::*$CDC27_{TAG5}$*-URA*	This study
yXF219	*yXF215 Δcdc27::*$CDC27_{TAG15}$*-URA*	This study
yXF220	*yXF215 Δcdc27::*$CDC27_{TAG451}$*-URA*	This study
yXF221	*yXF215 Δcdc27::*$CDC27_{TAG520}$*-URA*	This study
yXF217	*yXF215 Δcdc4::CDC4-URA3*	This study
yXF222	*yXF215 Δcdc4::*$CDC4_{TAG9}$*-URA3*	This study
yXF223	*yXF215 Δcdc4::*$CDC4_{TAG325}$*-URA3*	This study
yXF224	*yXF215 Δcdc4::*$CDC4_{TAG327}$*-URA3*	This study
yXF225	*yXF215 Δcdc4::*$CDC4_{TAG345}$*-URA3*	This study
yXF230	*yXF215 Δcdc27::*$CDC27_{TAG520}$*-BleoR Δcdc4::*$CDC4_{TAG9}$	This study
yXF226	*yXF215 Δalg1::*$ALG1_{TAG250}$*-URA3*	This study
yXF227	*yXF215 Δerg8::*$ERG8_{TAG401}$*-URA3*	This study
yXF228	*yXF215 Δrpn8::*$RPN8_{TAG7}$*-URA3*	This study
yXF229	*yXF215 Δgdi1::*$GDI1_{TAG5}$*-URA3*	This study
yXF237	*yXF223 [pXF469]*	This study
yXF238	*yXF223 [pRS423]*	This study
yXF285	*yXF223 [pXF500]*	This study
yXF286	*yXF221 [pXF469]*	This study
yXF320	*yXF221 [pRS423]*	This study
yXF321	*yXF221 [pXF500]*	This study
yXF239	*yXF215 Δ*P_{RPC11}*-RPC11::KanMX-*P_{GAL1}*-RPC11*	This study
yXF240	*yXF215 Δ*P_{SKP1}*-SKP1::KanMX-*P_{GAL1}*-SKP1*	This study
yXF241	*yXF215 Δ*P_{RPS3}*-RPS3::KanMX-*P_{GAL1}*-RPS3*	This study
yXF251	*yXF241 Δcdc4::*$CDC4_{TAG9}$*-URA3*	This study
yXF252	*yXF241 Δcdc4::*$CDC4_{TAG325}$*-URA3*	This study
yXF253	*yXF241 Δcdc27::*$CDC27_{TAG5}$*-URA*	This study
yXF254	*yXF241Δcdc27::*$CDC27_{TAG520}$*-URA*	This study
yXF290	*yXF241 Δcdc4::URA3-*$CDC4_{TAG4}$	This study
yXF291	*yXF241 Δcdc4::URA3-*$CDC4_{TAG5}$	This study
yXF292	*yXF241 Δcdc4::URA3-*$CDC4_{TAG6}$	This study
yXF293	*yXF241 Δcdc4::URA3-*$CDC4_{TAG7}$	This study
yXF294	*yXF241 Δcdc4::URA3-*$CDC4_{TAG9}$	This study
yXF295	*yXF241 Δcdc4::URA3-*$CDC4_{TAG12}$	This study
yXF296	*yXF241 Δcdc4::URA3-*$CDC4_{TAG13}$	This study
yXF297	*yXF241 Δcdc4::URA3-*$CDC4_{TAG14}$	This study
yXF298	*yXF241 Δcdc4::URA3-*$CDC4_{TAG15}$	This study
yXF299	*yXF241 Δcdc4::URA3-*$CDC4_{TAG16}$	This study
yXF300	*yXF241 Δcdc4::URA3-*$CDC4_{TAG18}$	This study

yXF301	*yXF241 Δcdc4::URA3-CDC4*$_{TAG19}$	This study
yXF302	*yXF241 Δcdc4::URA3-CDC4*$_{TAG20}$	This study
yXF303	*yXF241 Δcdc27::URA3-CDC27*$_{TAG4}$	This study
yXF304	*yXF241 Δcdc27::URA3-CDC27*$_{TAG5}$	This study
yXF305	*yXF241 Δcdc27::URA3-CDC27*$_{TAG6}$	This study
yXF306	*yXF241 Δcdc27::URA3-CDC27*$_{TAG7}$	This study
yXF307	*yXF241 Δcdc27::URA3-CDC27*$_{TAG8}$	This study
yXF308	*yXF241 Δcdc27::URA3-CDC27*$_{TAG9}$	This study
yXF309	*yXF241 Δcdc27::URA3-CDC27*$_{TAG10}$	This study
yXF310	*yXF241 Δcdc27::URA3-CDC27*$_{TAG11}$	This study
yXF311	*yXF241 Δcdc27::URA3-CDC27*$_{TAG12}$	This study
yXF312	*yXF241 Δcdc27::URA3-CDC27*$_{TAG13}$	This study
yXF313	*yXF241 Δcdc27::URA3-CDC27*$_{TAG14}$	This study
yXF314	*yXF241 Δcdc27::URA3-CDC27*$_{TAG15}$	This study
yXF315	*yXF241 Δcdc27::URA3-CDC27*$_{TAG16}$	This study
yXF316	*yXF241 Δcdc27::URA3-CDC27*$_{TAG17}$	This study
yXF317	*yXF241 Δcdc27::URA3-CDC27*$_{TAG18}$	This study
yXF318	*yXF241 Δcdc27::URA3-CDC27*$_{TAG19}$	This study
yXF319	*yXF241 Δcdc27::URA3-CDC27*$_{TAG20}$	This study
yXF243	*BY4741 Δcdc27::CDC27*^{G613D}*-KanMX*	This study
YFL009W^{ts}	*MATa his3Δ1 leu2Δ0 ura3Δ0 LYS2 MET5 can1Δ::LEU2-MFA1pr-HIS3 Δcdc4::CDC4*^{ts}*-URA3*	Junbiao Dai's lab
yXF289	*YFL009W*^{ts} *leu2-his3::BleoR*	This study
yXF244	*BY4741 Δnmd3::NMD3*^{ts}*-KanMX*	[2]
yXF287	*yXF222 Δupf1::KanMX*	This study
yXF288	*yXF222 Δupf2::KanMX*	This study
yXF281	*yXF307 YPL062W::tGeS-HIS3*	This study
yXF282	*yXF215 YPL062W::tGeS-HIS3*	This study
yXF448	*yXF215 Δnmd3::URA3-NMD3*	This study
yXF449	*yXF215 Δnmd3::URA3-NMD3*$_{TAG4}$	This study
yXF450	*yXF215 Δnmd3::URA3-NMD3*$_{TAG6}$	This study
yXF451	*yXF215 Δnmd3::URA3-NMD3*$_{TAG10}$	This study
yXF452	*yXF215 Δnmd3::URA3-NMD3*$_{TAG11}$	This study
yXF453	*yXF215 Δnmd3::URA3-NMD3*$_{TAG13}$	This study
yXF454	*yXF448 Δnmd3::NMD3~GFP-KanMX*	This study
yXF455	*yXF453 Δnmd3*$_{TAG13}$*::NMD3*$_{TAG13}$*~GFP-KanMX*	This study
yXF456	*MATa his3Δ1 leu2Δ0 met15Δ0 ura3Δ0 Δcdc27::CDC27*$_{TAG5}$*-URA3 [pXF509]*	This study
yXF457	*MATa his3Δ1 leu2Δ0 met15Δ0 ura3Δ0 Δcdc27::CDC27*$_{TAG5}$*-URA3 ΔP*$_{RPC11}$*-RPC11::KanMX-P*$_{GAL1}$*-RPC11 [pXF509]*	This study
E. coli		
DH5α	*F*$^{-}$ *80dlacZ M15 (lacZYA-argF) U169 recA1 endA1hsdR17(rk-, mk+) phoAsupE44 -thi-1 gyrA96 relA1*	Vazyme Cat number: C504-02

Sequence of customized plasmid used in this study

pXF231:TDH3 promoter, LeuOmeRS, CYC1 terminator, $tRNA_{CUA}^{Leu}$, SNR52 promoter

Note:The backbone is pRS315.

tcgcgcgtttcggtgatgacggtgaaaacctctgacacatgcagctcccggagacggtcacagcttgtctgtaagcggatgccgggagcagacaagcccgtcagg
gcgcgtcagcgggtgttggcgggtgtcggggctggcttaactatgcggcatcagagcagattgtactgagagtgcaccatatcgactacgtcgtaaggccgtttctgac
agagtaaaattcttgagggaactttcaccattatgggaaatggttcaagaaggtattgacttaaactccatcaaatggtcaggtcattgagtgttttttatttgttgtattttttttt
tttagagaaaatcctccaatatcaaattaggaatcgtagtttcatgattttctgttacacctaactttttgtgtggtgccctcctccttgtcaatattaatgttaaagtgcaattctttt
tccttatcacgttgagccattagtatcaatttgcttacctgtattcctttactatcctcctttttctccttcttgataaatgtatgtagattgcgtatatagtttcgtctaccctatgaaca
tattccattttgtaatttcgtgtcgtttctattatgaatttcatttataaagtttatgtacaaatatcataaaaaaagagaatctttttaagcaaggattttcttaacttcttcggcgac
agcatcaccgacttcggtggtactgttggaaccacctaaatcaccagttctgatacctgcatccaaaaccttttaactgcatcttcaatggccttaccttcttcaggcaagt
tcaatgacaatttcaacatcattgcagcagacaagatagtggcgatagggtcaaccttattctttggcaaatctggagcagaaccgtggcatggttcgtacaaaccaa
atgcggtgttcttgtctggcaaagaggccaaggacgcagatggcaacaaacccaaggaacctgggataacggaggcttcatcggagatgatatcaccaaacatgt
tgctggtgattataataccatttaggtgggttgggttcttaactaggatcatggcggcagaatcaatcaattgatgttgaaccttcaatgtagggaattcgttcttgatggtttc
ctccacagtttttctccataatcttgaagaggccaaaacattagctttatccaaggaccaaataggcaatggtggctcatgttgtagggccatgaaagcggccattcttgt
gattctttgcacttctggaacggtgtattgttcactatcccaagcgacaccatcaccatcgtcttcctttctcttaccaaagtaaatacctcccactaattctctgacaacaac
gaagtcagtacctttagcaaattgtggcttgattggagataagtctaaaagagagtcggatgcaaagttacatggtcttaagttggcgtacaattgaagttctttacggatt
tttagtaaaccttgttcaggtctaacactaccggtaccccatttaggaccacccacagcacctaacaaaacggcatcaaccttcttggaggcttccagcgcctcatctgg
aagtgggacacctgtagcatcgatagcagcaccaccaattaaatgattttcgaaatcgaacttgacattggaacgaacatcagaaatagctttaagaaccttaatggc
ttcggctgtgatttcttgaccaacgtggtcacctggcaaaacgacgatcttcttaggggcagacataggggcagacattagaatggtatatccttgaaatatatatatatat
tgctgaaatgtaaaaggtaagaaaagttagaaagtaagacgattgctaaccacctattggaaaaaacaataggtccttaaataatattgtcaacttcaagtattgtgat
gcaagcatttagtcatgaacgcttctctattctatatgaaaagccggttccggcctctcacctttcctttttctcccaatttttcagttgaaaaaggtatatgcgtcaggcgacct
ctgaaattaacaaaaaatttccagtcatcgaatttgattctgtgcgatagcgcccctgtgtgttctcgttatgttgaggaaaaaaataatggttgctaagagattcgaactct
tgcatcttacgatacctgagtattcccacagttaactgcggtcaagatatttcttgaatcaggcgccttagaccgctcggccaaacaaccaattacttgttgagaaataga
gtataattatcctataaatataacgtttttgaacacacatgaacaaggaagtacaggacaattgattttgaagagaatgtggattttgatgtaattgttgggattccatttttaa
taaggcaataatattaggtatgtggatatactagaagttctcctcgagggtcgatatgcggtgtgaaataccgcacagatgcgtaaggagaaaataccgcatcagga
aattgtaaacgttaatattttgttaaaattcgcgttaaatttttgttaaatcagctcattttttaaccaataggccgaaatcggcaaaatcccttataaatcaaaagaatagac
cgagatagggttgagtgttgttccagtttggaacaagagtccactattaaagaacgtggactccaacgtcaaagggcgaaaaaccgtctatcagggcgatggccca
ctacgtgaaccatcaccctaatcaagttttttggggtcgaggtgccgtaaagcactaaatcggaaccctaaagggagcccccgatttagagcttgacggggaaagcc
ggcgaacgtggcgagaaaggaagggaagaaagcgaaaggagcgggcgctagggcgctggcaagtgtagcggtcacgctgcgcgtaaccaccacacccgcc
gcgcttaatgcgccgctacagggcgcgtcgcgccattcgccattcaggctgcgcaactgttgggaagggcgatcggtgcgggcctcttcgctattacgccagctggc
gaagggggatgtgctgcaaggcgattaagttgggtaacgccagggttttcccagtcacgacgttgtaaaacgacggccagtgaattgtaatacgactcactatagg
gcgaattggagctcagtttatcattatcaatactcgccatttcaaagaatacgtaaataattaatagtagtgattttcctaactttatttagtcaaaaaattagccttttaattctg
ctgtaacccgtacatgcccaaaatagggggcgggttacacagaatatataacatcgtaggtgtctgggtgaacagtttattcctggcatccactaaatataatggagcc
cgctttttaagctggcatccagaaaaaaaaagaatcccagcaccaaaatattgttttcttcaccaaccatcagttcataggtccattctcttagcgcaactacagagaac
aggggcacaaacaggcaaaaaacgggcacaacctcaatggagtgatgcaacctgcctggagtaaatgatgacacaaggcaattgacccacgcatgtatctatct
cattttcttacaccttctattaccttctgctctctctgatttggaaaaagctgaaaaaaaaggttgaaaccagttccctgaaattattcccctacttgactaataagtatataaa
gacggtaggtattgattgtaattctgtaaatctatttcttaaacttcttaaattctacttttatagttagtcttttttttagttttaaaacaccagaacttagtttcgacggattctagaa
ctagtatggagatttccatggaagagcaataccgcccggaagagatagaatccaaagtacagcttcattgggatgagaagcgcacatttgaagtaaccgaagacg
agagcaaagagaagtattactgcctgtctttggaaccctatccttctggtcgactacacatgggccacgtacgtaactacaccatcggtgacgtgatcgcccgctacca
gcgtatgctgggcaaaaacgtcctgcagccgatcggctgggacgcgtttggtctgcctgcggaaggcgcggcggtgaaaaacaacaccgctccggcaccgtgga
cgtacgacaacatcgcgtatatgaaaaaccagctcaaaatgctgggctttggttatgactggagccgcgagctggcaacctgtacgccggaatactaccgttgggaa
cagaaattcttcaccgagctgtataaaaaaggcctggtatataagaagacttctgcggtcaactggtgcccgaacgaccagaccgtactggcgaacgaacaagttat
cgacggctgctgctggcgctgcgataccaaagttgaacgtaaagagatcccgcagtggtttatcaaaatcactgcttacgctgacgagctgctcaacgatctggataa
actggatcactggccagacaccgttaaaaccatgcagcgtaactggatcggtcgttccgaaggcgtggagatcaccttcaacgttaacgactatgacaacacgctg
accgtttacactacccgcccggacgcttttatgggttgtacctacctggcggtagctgcgggtcatccgctggcgcagaaagcggcggaaaataatcctgaactggcg
gcctttattgacgaatgccgtaacaccaaagttgccgaagctgaaatggcgacgatggagaaaaaaggcgtcgatactggctttaaagcggttcacccattaacgg
gcgaagaaattcccgtttgggcagcaaacttcgtattgatggagtacggcacgggcgcagttatggcggtaccggggcacgaccagcgcgactacgagtttgcctct
aaatacggcctgaacatcaaaccggttatcctggcagctgacggctctgagccagatctttctcagcaagccctgactgaaaaaggcgtgctgttcaactctggcgag
ttcaacggtcttgaccatgaagcggccttcaacgccatcgccgataaactgactgcgatgggcgttggcgagcgtaaattgaactaccgcctgcgcgactggggtgtt
tcccgtcagcgttactggggcgcgccgattccgatggtgacgctggaagacggtaccgtaatgccgaccccggacgaccagctgccggtgatcctgccggaggat
gtggtaatggacggcattaccagcccgattaaagcagatccggagtgggcgaaaactaccgttaacggtatgccagcactgcgtgaaaccgacactttcgacacct
ttatggagtcctcctggagatatgcgcgctacacttgcccgcagtacaaagaaggtatgctggattccgaagcggctaactactggctgccggtggatatcgctattggt
ggtattgaacacgccattatgggtctgctctacttccgcttcttccacaaactgatgcgtgatgcaggcatggtgaactctgacgaaccagcgaaacagttgctgtgtca
gggtatggtgctggcagatgccttctactatgttggcgaaaacggcgaacgtaactgggtttccccggttgatgctatcgttgaacgtgacgagaaaggccgtatcgtg
aaagcgaaagatgcggcaggccatgaactggtttataccggcatgagcaaaatgtccaagtcgaagaacaacggtatcgacccgcaggtgatggttgaacgttac
ggcgcggacaccgttcgtctgtttatgatgtttgcttctccggctgatatgactctcgaatggcaggaatccggtgtggaaggggctaaccgcttcctgaaacgtgtctgg
aaactggtttacgagcacacagcaaaaggtgatgttgcggcactgaacgttgatgcgctgactgaaaatcagaaagcgctgcgtcgcgatgtgcataaaacgatcg
ctaaagtgaccgatgatatcggccgtcgtcagaccttcaacaccgcaattgcggcgattatggagctgatgaacaaactggcgaaagcaccaaccgatggcgagc
aggatcgcgctctgatgcaggaagcactgctggccgttgtccgtatgcttaacccgttcaccccgcacatctgcttcacgctgtggcaggaactgaaaggcgaaggc

gatatcgacaacgcgccgtggccggttgctgacgaaaaagcgatggtggaagactccacgctggtcgtggtgcaggttaacggtaaggtccgtgccaaaatcacc
gttccggtggacgcaacggaagaacaggttcgcgaacgtgctggccaggaacatctggtagcaaaatatcttgatggcgttactgtacgtaaagtgatttacgtacca
ggtaaactcctcaatctggtcgttggctaacatatgtcacgcttacattcacgccctccccccacatccgctctaaccgaaaaggaaggagttagacaacctgaagtct
aggtccctatttattttttatagttatgttagtattaagaacgttatttatatttcaaatttttcttttttttctgtacagacgcgtgtacgcatgtaacattatactgaaaaccttgcttg
agaaggttttgggacgctcgaaggctttaatttgcggccggtacccctggaaggtgccactcccactgtcctttcctaataaaatgaggaaattgcatcgcattgtctgag
taggtgtcattctattctgggggggtggggtggggcaggacagcaagggggaggattgggaagacaatagcaggcatgctggggatgcggtgggctctatggcttctg
aggcggaaagaaccagctggggctctagggggtatccccacgcgccctgtagcggcgcattaagcgcggcgggtgtggtggttacgcgcagcgtgaccgctaca
cttgccagcgccctagcgcccgcactagagacgtcccgcggggcggctctagacataaaaaacaaaaaaatacccggagcgggacttgaacccgcacagcgc
gaacgccgagggatttagaatcccttgtgtctaccgattccaccatccgggctatttaattgttgaagaaagagtatactacataacacatatacaattgaaaaagagg
ctagcagcttaccggtcgggatggctgcaggatcatttatctttcactgcggagaagtttcgaacgccgaaacatgcgcaccaactttcacttctacagcgtttgaccaa
aatcttttgaacagaacattgtagggtgtgaaaaaatgcgcacctttaccgctagcccaagagggcactacaaaatctagagttgtacttcaaacgtacatgtaatcac
cttgtatatactcgaaagaaaacatcaagtttctgtataaatatgagtgaaagcataatcatacattatcttttcaaagactgcaggaattcgatatcaagcttatcgatac
cgtcgacctcgagggggggcccggtacccagcttttgttccctttagtgagggttaattccgagcttggcgtaatcatggtcatagctgtttcctgtgtgaaattgttatccgc
tcacaattccacacaacataggagccggaagcataaagtgtaaagcctggggtgcctaatgagtgaggtaactcacattaattgcgttgcgctcactgcccgctttcc
agtcgggaaacctgtcgtgccagctgcattaatgaatcggccaacgcgcggggagaggcggtttgcgtattgggcgctcttccgcttcctcgctcactgactcgctgcg
ctcggtcgttcggctgcggcgagcggtatcagctcactcaaaggcggtaatacggttatccacagaatcaggggataacgcaggaaagaacatgtgagcaaaag
gccagcaaaaggccaggaaccgtaaaaaggccgcgttgctggcgtttttccataggctcggcccccctgacgagcatcacaaaaatcgacgctcaagtcagaggt
ggcgaaacccgacaggactataaagataccaggcgttcccccctggaagctccctcgtgcgctctcctgttccgaccctgccgcttaccggatacctgtccgcctttct
cccttcgggaagcgtggcgctttctcaatgctcacgctgtaggtatctcagttcggtgtaggtcgttcgctccaagctgggctgtgtgcacgaaccccccgttcagcccga
ccgctgcgccttatccggtaactatcgtcttgagtccaacccggtaagacacgacttatcgccactggcagcagccactggtaacaggattagcagagcgaggtatgt
aggcggtgctacagagttcttgaagtggtggcctaactacggctacactagaaggacagtatttggtatctgcgctctgctgaagccagttaccttcggaaaaagagtt
ggtagctcttgatccggcaaacaaaccaccgctggtagcggtggtttttttgtttgcaagcagcagattacgcgcagaaaaaaaggatctcaagaagatcctttgatctt
ttctacggggtctgacgctcagtggaacgaaaactcacgttaagggattttggtcatgagattatcaaaaaggatcttcacctagatccttttaaattaaaaatgaagtttt
aaatcaatctaaagtatatatgagtaaacttggtctgacagttaccaatgcttaatcagtgaggcacctatctcagcgatctgtctatttcgttcatccatagttgcctgactg
cccgtcgtgtagataactacgatacgggagggcttaccatctggccccagtgctgcaatgataccgcgagacccacgctcaccggctccagatttatcagcaataaa
ccagccagccggaagggccgagcgcagaagtggtcctgcaactttatccgcctccatccagtctattaattgttgccgggaagctagagtaagtagttcgccagttaa
tagtttgcgcaacgttgttgccattgctacaggcatcgtggtgtcacgctcgtcgtttggtatggcttcattcagctccggttcccaacgatcaaggcgagttacatgatccc
ccatgttgtgaaaaaaagcggttagctccttcggtcctccgatcgttgtcagaagtaagttggccgcagtgttatcactcatggttatggcagcactgcataattctcttact
gtcatgccatccgtaagatgcttttctgtgactggtgagtactcaaccaagtcattctgagaatagtgtatgcggcgaccgagttgctcttgcccggcgtcaatacgggat
aataccgcgccacatagcagaactttaaaagtgctcatcattggaaaacgttcttcggggcgaaaactctcaaggatcttaccgctgttgagatccagttcgatgtaac
ccactcgtgcacccaactgatcttcagcatcttttactttcaccagcgtttctgggtgagcaaaaacaggaaggcaaaatgccgcaaaaaagggaataagggcgac
acggaaatgttgaatactcatactcttcctttttcaatattattgaagcatttatcagggttattgtctcatgagcggatacatatttgaatgtatttagaaaaataaacaaatag
gggttccgcgcacatttccccgaaaagtgccacctgggtccttttcatcacgtgctataaaaataattataatttaaattttttaatataaatatataaattaaaaatagaaa
gtaaaaaaagaaattaaagaaaaaatagtttttgttttccgaagatgtaaaagactctagggggatcgccaacaaatactaccttttatcttgctcttcctgctctcaggta
ttaatgccgaattgtttcatcttgtctgtgtagaagaccacacacgaaaatcctgtgattttacattttacttatcgttaatcgaatgtatatctatttaatctgcttttcttgtctaata
aatatatatgtaaagtacgctttttgttgaaattttttaaacctttgtttattttttttttcttcattccgtaactcttctaccttctttatttactttctaaaatccaaatacaaaacataaaa
ataaataaacacagagtaaattcccaaattattccatcattaaaagatacgaggcgcgtgtaagttacaggcaagcgatccgtcctaagaaaccattattatcatgac
attaacctataaaaataggcgtatcacgaggccctttcgt

pXF509:TDH3 promoter, LeuOmeRS, CYC1 terminator, $tRNA_{CUA}^{Leu}$, SNR52 promoter

Note:The backbone is pRS313

ggagaaaataccgcatcaggaaattgtaaacgttaatattttgttaaaattcgcgttaaatttttgttaaatcagctcattttttaaccaataggccgaaatcggcaaaatcc
cttataaatcaaaagaatagaccgagatagggttgagtgttgttccagtttggaacaagagtccactattaaagaacgtggactccaacgtcaaagggcgaaaaac
cgtctatcagggcgatggcccactacgtgaaccatcaccctaatcaagttttttggggtcgaggtgccgtaaagcactaaatcggaaccctaaagggagcccccgatt
tagagcttgacggggaaagccggcgaacgtggcgagaaaggaagggaagaaagcgaaaggagcgggcgctagggcgctggcaagtgtagcggtcacgctg
cgcgtaaccaccacacccgccgcgcttaatgcgccgctacagggcgcgtcgcgccattcgccattcaggctgcgcaactgttgggaagggcgatcggtgcgggcc
tcttcgctattacgccagctggcgaaggggggatgtgctgcaaggcgattaagttgggtaacgccagggttttcccagtcacgacgttgtaaaacgacggccagtga
attgtaatacgactcactatagggcgaattggagctcagtttatcattatcaatactcgccatttcaaagaatacgtaaataattaatagtagtgattttcctaactttatttagt
caaaaaattagccttttaattctgctgtaacccgtacatgcccaaaatagggggcgggttacacagaatatataacatcgtaggtgtctgggtgaacagtttattcctggc
atccactaaatataatggagcccgctttttaagctggcatccagaaaaaaaaagaatcccagcaccaaaatattgttttcttcaccaaccatcagttcataggtccattct
cttagcgcaactacagagaacaggggcacaaacaggcaaaaaacgggcacaacctcaatggagtgatgcaacctgcctggagtaaatgatgacacaaggca
attgacccacgcatgtatctatctcattttcttacaccttctattaccttctgctctctctgatttggaaaaagctgaaaaaa
tagttttaaaacaccagaacttagtttcgaaaggttgaaaccagttccctgaaattattcccctacttgactaataagtatataaagacggtaggtattgattgtaattctgta
aatctatttcttaaacttcttaaattctacttttatagttagtcttttttttagttttaaaacaccagaacttagtttcgacggattctagaactagtatggagatttccatggaagag
caataccgcccggaagagatagaatccaaagtacagcttcattgggatgagaagcgcacatttgaagtaaccgaagacgagagcaaagagaagtattactgcct
gtctttggaaccctatccttctggtcgactacacatgggccacgtacgtaactacaccatcggtgacgtgatcgcccgctaccagcgtatgctgggcaaaaacgtcctg
cagccgatcggctgggacgcgtttggtctgcctgcggaaggcgcggcggtgaaaaacaacaccgctccggcaccgtggacgtacgacaacatcgcgtatatgaa
aaaccagctcaaaatgctgggctttggttatgactggagccgcgagctggcaacctgtacgccggaatactaccgttgggaacagaaattcttcaccgagctgtataa

aaaaggcctggtatataagaagacttctgcggtcaactggtgcccgaacgaccagaccgtactggcgaacgaacaagttatcgacggctgctgctggcgctgcgat
accaaagttgaacgtaaagagatcccgcagtggtttatcaaaatcactgcttacgctgacgagctgctcaacgatctggataaactggatcactggccagacaccgtt
aaaaccatgcagcgtaactggatcggtcgttccgaaggcgtggagatcaccttcaacgttaacgactatgacaacacgctgaccgtttacactacccgcccggacg
cttttatgggttgtacctacctggcggtagctgcgggtcatccgctggcgcagaaagcggcggaaaataatcctgaactggcggcctttattgacgaatgccgtaacac
caaagttgccgaagctgaaatggcgacgatggagaaaaaaggcgtcgatactggctttaaagcggttcacccattaacgggcgaagaaattcccgtttgggcagc
aaacttcgtattgatggagtacggcacgggcgcagttatggcggtaccggggcacgaccagcgcgactacgagtttgcctctaaatacggcctgaacatcaaaccg
gttatcctggcagctgacggctctgagccagatctttctcagcaagccctgactgaaaaaggcgtgctgttcaactctggcgagttcaacggtcttgaccatgaagcgg
ccttcaacgccatcgccgataaactgactgcgatgggcgttggcgagcgtaaattgaactaccgcctgcgcgactggggtgtttcccgtcagcgttactggggcgcgc
cgattccgatggtgacgctggaagacggtaccgtaatgccgaccccggacgaccagctgccggtgatcctgccggaggatgtggtaatggacggcattaccagcc
cgattaaagcagatccggagtgggcgaaaactaccgttaacggtatgccagcactgcgtgaaaccgacactttcgacacctttatggagtcctcctggagatatgcg
cgctacacttgcccgcagtacaaagaaggtatgctggattccgaagcggctaactactggctgccggtggatatcgctattggtggtattgaacacgccattatgggtct
gctctacttccgcttcttccacaaactgatgcgtgatgcaggcatggtgaactctgacgaaccagcgaaacagttgctgtgtcagggtatggtgctggcagatgccttct
actatgttggcgaaaacggcgaacgtaactgggtttccccggttgatgctatcgttgaacgtgacgagaaaggccgtatcgtgaaagcgaaagatgcggcaggcca
tgaactggtttataccggcatgagcaaaatgtccaagtcgaagaacaacggtatcgacccgcaggtgatggttgaacgttacggcgcggacaccgttcgtctgtttat
gatgtttgcttctccggctgatatgactctcgaatggcaggaatccggtgtggaaggggctaaccgcttcctgaaacgtgtctggaaactggtttacgagcacacagca
aaaggtgatgttgcggcactgaacgttgatgcgctgactgaaaatcagaaagcgctgcgtcgcgatgtgcataaaacgatcgctaaagtgaccgatgatatcggcc
gtcgtcagaccttcaacaccgcaattgcggcgattatggagctgatgaacaaactggcgaaagcaccaaccgatggcgagcaggatcgcgctctgatgcaggaa
gcactgctggccgttgtccgtatgcttaacccgttcaccccgcacatctgcttcacgctgtggcaggaactgaaaggcgaaggcgatatcgacaacgcgccgtggcc
ggttgctgacgaaaaagcgatggtggaagactccacgctggtcgtggtgcaggttaacggtaaggtccgtgccaaaatcaccgttccggtggacgcaacggaaga
acaggttcgcgaacgtgctggccaggaacatctggtagcaaaatatcttgatggcgttactgtacgtaaagtgatttacgtaccaggtaaactcctcaatctggtcgttg
gctaacatatgtcacgcttacattcacgccctccccccacatccgctctaaccgaaaaggaaggagttagacaacctgaagtctaggtccctatttatttttttatagttatg
ttagtattaagaacgttatttatatttcaaatttttcttttttttctgtacagacgcgtgtacgcatgtaacattatactgaaaaccttgcttgagaaggttttgggacgctcgaagg
ctttaatttgcggccggtacccctggaaggtgccactcccactgtcctttcctaataaaatgaggaaattgcatcgcattgtctgagtaggtgtcattctattctggggggtg
gggtggggcaggacagcaagggggaggattgggaagacaatagcaggcatgctggggatgcggtgggctctatggcttctgaggcggaaagaaccagctggg
gctctagggggtatccccacgcgccctgtagcggcgcattaagcgcggcgggtgtggtggttacgcgcagcgtgaccgctacacttgccagcgccctagcgcccgc
actagagacgtcccgcggggcggctctagacataaaaaacaaaaaaatacccggagcgggacttgaacccgcacagcgcgaacgccgagggatttagaatcc
cttgtgtctaccgattccaccatccgggctatttaattgttgaagaaagagtatactacataacacatatacaattgaaaaagaggctagcagcttaccggtcgggatgg
ctgcaggatcatttatctttcactgcggagaagtttcgaacgccgaaacatgcgcaccaactttcacttctacagcgtttgaccaaaatcttttgaacagaacattgtagg
gtgtgaaaaaatgcgcacctttaccgctagcccaagagggcactacaaaatctagagttgtacttcaaacgtacatgtaatcaccttgtatatactcgaaagaaaaca
tcaagtttctgtataaatatgagtgaaagcataatcatacattatcttttcaaagactgcaggaattcgatatcaagcttatcgataccgtcgacctcgagggggggcccg
gtacccagcttttgttccctttagtgagggttaattccgagcttggcgtaatcatggtcatagctgtttcctgtgtgaaattgttatccgctcacaattccacacaacatacgag
ccggaagcataaagtgtaaagcctggggtgcctaatgagtgaggtaactcacattaattgcgttgcgctcactgcccgctttccagtcgggaaacctgtcgtgccagct
gcattaatgaatcggccaacgcgcggggagaggcggtttgcgtattgggcgctcttccgcttcctcgctcactgactcgctgcgctcggtcgttcggctgcggcgagcg
gtatcagctcactcaaaggcggtaatacggttatccacagaatcaggggataacgcaggaaagaacatgtgagcaaaaggccagcaaaaggccaggaaccgt
aaaaaggccgcgttgctggcgtttttccataggctcggcccccctgacgagcatcacaaaaatcgacgctcaagtcagaggtggcgaaacccgacaggactataa
agataccaggcgtttccccctggaagctccctcgtgcgctctcctgttccgaccctgccgcttaccggatacctgtccgcctttctcccttcgggaagcgtggcgctttctc
aatgctcacgctgtaggtatctcagttcggtgtaggtcgttcgctccaagctgggctgtgtgcacgaaccccccgttcagcccgaccgctgcgccttatccggtaactatc
gtcttgagtccaacccggtaagacacgacttatcgccactggcagcagccactggtaacaggattagcagagcgaggtatgtaggcggtgctacagagttcttgaag
tggtggcctaactacggctacactagaaggacagtatttggtatctgcgctctgctgaagccagttaccttcggaaaaagagttggtagctcttgatccggcaaacaaa
ccaccgctggtagcggtggtttttttgtttgcaagcagcagattacgcgcagaaaaaaaggatctcaagaagatcctttgatcttttctacggggtctgacgctcagtgga
acgaaaactcacgttaagggattttggtcatgagattatcaaaaaggatcttcacctagatccttttaaattaaaaatgaagttttaaatcaatctaaagtatatatgagta
aacttggtctgacagttaccaatgcttaatcagtgaggcacctatctcagcgatctgtctatttcgttcatccatagttgcctgactgcccgtcgtgtagataactacgatac
gggagggcttaccatctggccccagtgctgcaatgataccgcgagacccacgctcaccggctccagatttatcagcaataaaccagccagccggaagggccgag
cgcagaagtggtcctgcaactttatccgcctccatccagtctattaattgttgccgggaagctagagtaagtagttcgccagttaatagtttgcgcaacgttgttgccattgc
tacaggcatcgtggtgtcacgctcgtcgtttggtatggcttcattcagctccggttcccaacgatcaaggcgagttacatgatcccccatgttgtgaaaaaaagcggttag
ctccttcggtcctccgatcgttgtcagaagtaagttggccgcagtgttatcactcatggttatggcagcactgcataattctcttactgtcatgccatccgtaagatgcttttctg
tgactggtgagtactcaaccaagtcattctgagaatagtgtatgcggcgaccgagttgctcttgcccggcgtcaatacgggataataccgcgccacatagcagaacttt
aaaagtgctcatcattggaaaacgttcttcggggcgaaaactctcaaggatcttaccgctgttgagatccagttcgatgtaacccactcgtgcacccaactgatcttcag
catcttttactttcaccagcgtttctgggtgagcaaaaacaggaaggcaaaatgccgcaaaaaagggaataagggcgacacggaaatgttgaatactcatactcttc
ctttttcaatattattgaagcatttatcagggttattgtctcatgagcggatacatatttgaatgtatttagaaaaataaacaaataggggttccgcgcacatttccccgaaaa
gtgccacctgggtccttttcatcacgtgctataaaaataattataatttaaatttttaatataaatatataaattaaaaatagaaagtaaaaaaagaaattaaagaaaaaa
tagtttttgttttccgaagatgtaaaagactctagggggatcgccaacaaatactaccttttatcttgctcttcctgctctcaggtattaatgccgaattgtttcatcttgtctgtgt
agaagaccacacacgaaaatcctgtgattttacattttacttatcgttaatcgaatgtatatctatttaatctgcttttcttgtctaataaatatatatgtaaagtacgctttttgttg
aaattttttaaacctttgtttattttttttttcttcattccgtaactcttctaccttctttatttactttctaaaatccaaatacaaaacataaaaataaataaacacagagtaaattccc

aaattattccatcattaaaagatacgaggcgcgtgtaagttacaggcaagcgatccgtcctaagaaaccattattatcatgacattaacctataaaaataggcgtatca
cgaggccctttcgttcgcgcgtttcggtgatgacggtgaaaacctctgacacatgcagctcccggagacggtcacagcttgtctgtaagcggatgccgggagcagac
aagcccgtcagggcgcgtcagcgggtgttggcgggtgtcggggctggcttaactatgcggcatcagagcagattgtactcacaccgcatagatccgtcgagttcaag
agaaaaaaaaagaaaaagcaaaaagaaaaaaggaaagcgcgcctcgttcagaatgacacgtatagaatgatgcattaccttgtcatcttcagtatcatactgttcg
tatacatacttactgacattcataggtatacatatatacacatgtatatatatcgtatgctgcagctttaaataatcggtgtcactacataagaacacctttggtggagggaa
catcgttggtaccattgggcgaggtggcttctcttatggcaaccgcaagagccttgaacgcactctcactacggtgatgatcattcttgcctcgcagacaatcaacgtgg
agggtaattctgctagcctctgcaaagctttcaagaaaatgcgggatcatctcgcaagagagatctcctactttctccctttgcaaaccaagttcgacaactgcgtacgg
cctgttcgaaagatctaccaccgctctggaaagtgcctcatccaaaggcgcaaatcctgatccaaacctttttactccacgcgccagtagggcctctttaaaagcttgac
cgagagcaatcccgcagtcttcagtggtgtgatggtcgtctatgtgtaagtcaccaatgcactcaacgattagcgaccagccggaatgcttggccagagcatgtatcat
atggtccagaaaccctatacctgtgtggacgttaatcacttgcgattgtgtggcctgttctgctactgcttctgcctctttttctgggaagatcgagtgctctatcgctaggga
ccacccttaaagagatcgcaatctgaatcttggtttcatttgtaatacgctttactagggctttctgctctgtcatctttgccttcgtttatcttgcctgctcatttttagtatattcttc
gaagaaatcacattactttatataatgtataattcattatgtgataatgccaatcgctaagaaaaaaaaagagtcatccgctaggggaaaaaaaaaaatgaaaatcat
taccgaggcataaaaaaatatagagtgtactagaggaggccaagagtaatagaaaaagaaaattgcgggaaaggactgtgttatgacttccctgactaatgc

pXF484-489: CDC4 promoter, CDC4 (TAG codon), CDC4 terminator

Note: The blue corresponding codon were individually replaced with the TAG stop codon via site-directed mutagenesis.

gacgaaagggcctcgtgatacgcctatttttataggttaatgtcatgataataatggtttcttaggacggatcgcttgcctgtaacttacacgcgcctcgtatcttttaatgatg
gaataatttgggaatttactctgtgtttatttatttttatgttttgtatttggattttagaaagtaaataaagaaggtagaagagttacggaatgaagaaaaaaaaaataaacaa
aggtttaaaaaatttcaacaaaaagcgtactttacatatatatttattagacaagaaaagcagattaaatagatatacattcgattaacgataagtaaaatgtaaaatca
caggattttcgtgtgtggtcttctacacagacaagatgaaacaattcggcattaatacctgagagcaggaagagcaagataaaaggtagtatttgttggcgatcccct
agagtcttttacatcttcggaaaacaaaaactattttttctttaatttctttttttactttctatttttaatttatatatttatattaaaaaatttaaattataattatttttatagcacgtgatg
aaaaggacccaggtggcacttttcggggaaatgtgcgcggaacccctatttgtttatttttctaaatacattcaaatatgtatccgctcatgagacaataaccctgataaat
gcttcaataatattgaaaaaggaagagtatgagtattcaacatttccgtgtcgcccttattcccttttttgcggcattttgccttcctgtttttgctcacccagaaacgctggtga
aagtaaaagatgctgaagatcagttgggtgcacgagtgggttacatcgaactggatctcaacagcggtaagatccttgagagttttcgccccgaagaacgttttccaat
gatgagcacttttaaagttctgctatgtggcgcggtattatcccgtattgacgccgggcaagagcaactcggtcgccgcatacactattctcagaatgacttggttgagta
ctcaccagtcacagaaaagcatcttacggatggcatgacagtaagagaattatgcagtgctgccataaccatgagtgataacactgcggccaacttacttctgacaa
cgatcggaggaccgaaggagctaaccgcttttttgcacaacatgggggatcatgtaactcgccttgatcgttgggaaccggagctgaatgaagccataccaaacga
cgagcgtgacaccacgatgcctgtagcaatggcaacaacgttgcgcaaactattaactggcgaactacttactctagcttcccggcaacaattaatagactggatgg
aggcggataaagttgcaggaccacttctgcgctcggcccttccggctggctggtttattgctgataaatctggagccggtgagcgtgggtctcgcggtatcattgcagca
ctggggccagatggtaagccctcccgtatcgtagttatctacacgacggggagtcaggcaactatggatgaacgaaatagacagatcgctgagataggtgcctcact
gattaagcattggtaactgtcagaccaagtttactcatatatactttagattgatttaaaacttcatttttaatttaaaaggatctaggtgaagatcctttttgataatctcatgac
caaaatcccttaacgtgagttttcgttccactgagcgtcagaccccgtagaaaagatcaaaggatcttcttgagatcctttttttctgcgcgtaatctgctgcttgcaaacaa
aaaaaccaccgctaccagcggtggtttgtttgccggatcaagagctaccaactctttttccgaaggtaactggcttcagcagagcgcagataccaaatactgtccttcta
gtgtagccgtagttaggccaccacttcaagaactctgtagcaccgcctacatacctcgctctgctaatcctgttaccagtggctgctgccagtggcgataagtcgtgtctt
accgggttggactcaagacgatagttaccggataaggcgcagcggtcgggctgaacggggggttcgtgcacacagcccagcttggagcgaacgacctacaccg
aactgagatacctacagcgtgagctatgagaaagcgccacgcttcccgaagggagaaaggcggacaggtatccggtaagcggcagggtcggaacaggagag
cgcacgagggagcttccagggggaaacgcctggtatctttatagtcctgtcgggtttcgccacctctgacttgagcgtcgatttttgtgatgctcgtcaggggggcggag
cctatggaaaaacgccagcaacgcggcctttttacggttcctggccttttgctggccttttgctcacatgttctttcctgcgttatcccctgattctgtggataaccgtattaccg
cctttgagtgagctgataccgctcgccgcagccgaacgaccgagcgcagcgagtcagtgagcgaggaagcggaagagcgcccaatacgcaaaccgcctctccc
cgcgcgttggccgattcattaatgcagctggcacgacaggtttcccgactggaaagcgggcagtgagcgcaacgcaattaatgtgagttacctcactcattaggcac
cccaggctttacactttatgcttccggctcctatgttgtgtggaattgtgagcggataacaatttcacacaggaaacagctatgaccatgattacgccaagcgcgcaatta
accctcactaaagggaacaaaagctggagctccaccgcggtggcggccgctctagaactagtggatcttccagtcagtagtcagtagtcagttgatgtagtgctggtc
tggtattcatcatcaaaaacagccttccagccagaaggcacttgaggaggattacttttacttgagccattactttaaaatgttttgggtttttttatcgactctcttgtagtgag
tgataaaagcaattcagaaagagtcttttgattatcatgactaaagtaaactttttcttccagctgccggctcggtcaggtggacgaaacaaaaaagcctgttgaaataa
tactgaatacgttaaaacgtcacagcgcttgtatatcttgtgagagaaaggctattaccactgttatatgaatatagattgctcaacaaagcgtgtctttttctgtgtgcttaa
aatatagtgtctttttttgggaaaaaaacgtttatttacagtattctcttcttcttctcctcattcaacttgttgcggttcgaagaaaattacgcataaagaatcaaagaaaggca
aaaattacgctgtacgatggggtcgtttcccttagctgagtttccattacgtgatatccctgttccttatagctaccgtgtgtctggcggtatagcttcctcaggtagtgttactg
cgcttgttactgccgctggcactcatcgaaactcgtccacggctaagacagttgagacagaagacggcgaagaagatatcgatgagtatcagaggaaaagagca
gctggttctggcgaatccactcctgaacgcagtgatttcaaaagggtaaaacatgataatcacaaaaccctccatccagttaacttacagaacaccggtgcagcgtct
gtggataatgacggtctgcacaatttaacagatatatccaacgatgcagaaaaacttttgatgtctgtggatgatggttctgccgcaccttctacattgagtgtaaacatgg
gagtggcatctcataatgttgctgctcccactaccgtcaatgcggcaacaataactggcagtgatgttagtaacaatgttaatagtgctactattaacaatcctatggagg
aaggagcgctgccgttatcacccactgcttcctctccaggtaccacaactcctttagctaaaactacgaaaactatcaacaacaataataatatcgccgatttgataga
atccaaagattctataatctcccctgaatacctttctgatgagattttcagcgcaataaacaataatctccctcacgcatacttcaaaaatttattatttagattagttgccaac
atggataggagtgaactatccgacttggggactttaatcaaggataatttaaagagggacctaataacgtctttgccttttgaaataagtttgaaaattttcaattatttgca
attcgaggatattataaattcccttggggtctcccaaaattggaacaaaataattagaaaatctacatcgttgtggaaaaaacttctgatatcggaaaatttgtgagccc
aaagggttttaattctctcaatctcaaactctcccaaaaatacccaaaactctcacaacaagatcgccttagattatcttttctggagaatatattcattttaaaaaattggta
caatcccaagtttgtaccacaaaggaccacgttaagaggccatatgacgagtgttattacgtgcttgcaatttgaagataattatgtcattacgggggctgatgacaaaa

tgatcagagtttatgattcgataaacaagaaatttcttctacaactatcaggtcatgatggtggggtttgggcattgaagtatgcccatggcggtattttagtcagcggttcta
cagacagaacggtgcgagtttgggatattaagaaaggttgttgtacccatgtgtttaaaggtcataactctacggtgaggtgcctagatatagtagaatataaaaatatc
aagtacattgttactggttcgagagataacactttgcacgtttggaaattgcccaaggagtcctccgttcctgatcatggggaagaacatgattatccattagtctttcatac
ccctgaggagaacccatatttgttggtgttttaagaggacatatggcatctgtaagaactgtctcaggccacggtaatattgtcgttagtggctcctatgataatacactga
ttgtgtgggatgttgcgcaaatgaaatgtttgtatattttaagtggacatacggatcgtatttattcgacaatctacgatcatgaaagaaaaaggtgcatctctgccagtatg
gataccactattagaatttgggatttggaaaatatatggaataatggagaatgttcctacgcaacaaattcagcatcgccatgcgccaaaatacttggtgctatgtacact
ttgcagggtcatacagctttggtcggtttattaagattatccgacaaatttttggtcagtgccgctgcagacggttcaataaggggttgggacgcaaacgactactctaga
aaattttcctaccatcataccaatttgagtgcaattaccacattttatgtatcggataatattttggtgagtggatcggaaaatcagttcaacatctataatctacggagtggg
aaattggtccacgcaaatattctaaaagatgctgatcagatttggtcggttaattttaagggcaaaacacttgttgcagcagttgaaaaagatggacagagctttttagaa
attctggatttcagcaaagcttcaaaaattaactacgttagcaatcccgtaaactcctcgtcgtcgtctttggaatccatttctacttctttgggtctaacgaggacaactata
ataccatcgtcgggttgacctttcccagagaataagcattgactcatacttagataatatagcttaataagtagttatataatcagtaaaaaagtacaataacaacttcgt
acattttattgaatataaactgcagctaaactgcttgtatgttcaattttaattgtgtttacaaaaagggtgccgtttattaattaatgtttcttccctgaaaatatggaaagtaca
agtttttagttgagaagggtttaagaaagttttgaaaatgatctaaaaaaatataaaagcaatcaaagaaataaaagctggaaaaatgcgtaataaccgaagtgacc
tgtgcatcacttctcacgtctgaaccaagatagctggatgtacccaattcgccctatagtgagtcgtattacgcgcgctcactggccgtcgttttacaacgtcgtgactggg
aaaaccctggcgttacccaacttaatcgccttgcagcacatccccctttcgccagctggcgtaatagcgaagaggcccgcaccgatcgcccttcccaacagttgcgc
agcctgaatggcgaatggcgcgacgcgccctgtagcggcgcattaagcgcggcgggtgtggtggttacgcgcagcgtgaccgctacacttgccagcgccctagcg
cccgctcctttcgctttcttcccttcctttctcgccacgttcgccggctttccccgtcaagctctaaatcgggggctccctttagggttccgatttagtgctttacggcacctcga
ccccaaaaaacttgattagggtgatggttcacgtagtgggccatcgccctgatagacggtttttcgccctttgacgttggagtccacgttctttaatagtggactcttgttcca
aactggaacaacactcaaccctatctcggtctattcttttgatttataagggattttgccgatttcggcctattggttaaaaaatgagctgatttaacaaaaatttaacgcgaa
ttttaacaaaatattaacgtttacaatttcctgatgcggtattttctccttacgcatctgtgcggtatttcacaccgcatagatccgtcgagttcaagagaaaaaaaaagaaa
aagcaaaaagaaaaaaggaaagcgcgcctcgttcagaatgacacgtatagaatgatgcattaccttgtcatcttcagtatcatactgttcgtatacatacttactgacat
tcataggtatacatatatacacatgtatatatatcgtatgctgcagctttaaataatcggtgtcactacataagaacacctttggtggagggaacatcgttggtaccattgg
gcgaggtggcttctcttatggcaaccgcaagagccttgaacgcactctcactacggtgatgatcattcttgcctcgcagacaatcaacgtggagggtaattctgctagcc
tctgcaaagctttcaagaaaatgcgggatcatctcgcaagagagatctcctactttctccctttgcaaaccaagttcgacaactgcgtacggcctgttcgaaagatctac
caccgctctggaaagtgcctcatccaaaggcgcaaatcctgatccaaaccttttttactccacgcgccagtagggcctctttaaaagcttgaccgagagcaatcccgca
gtcttcagtggtgtgatggtcgtctatgtgtaagtcaccaatgcactcaacgattagcgaccagccggaatgcttggccagagcatgtatcatatggtccagaaaccta
tacctgtgtggacgttaatcacttgcgattgtgtggcctgttctgctactgcttctgcctctttttctgggaagatcgagtgctctatcgctaggggaccaccctttaaagagat
cgcaatctgaatcttggtttcatttgtaatacgctttactagggctttctgctctgtcatctttgccttcgtttatcttgcctgctcatttttttagtatattcttcgaagaaatcacattact
ttatataatgtataattcattatgtgataatgccaatcgctaagaaaaaaaaagagtcatccgctaggggaaaaaaaaaaaatgaaaatcattaccgaggcataaaaa
aatatagagtgtactagaggaggccaagagtaatagaaaaagaaaattgcgggaaaggactgtgttatgacttccctgactaatgccgtgttcaaacgatacctggc
agtgactcctagcgctcaccaagctcttaaaacgggaatttatggtgcactctcagtacaatctgctctgatgccgcatagttaagccagccccgacacccgccaaca
cgcgctgacgcgccctgacgggcttgtctgctcccggcatccgcttacagacaagctgtgaccgtctccgggagctgcatgtgtcagaggttttcaccgtcatcaccga
aacgcgcga

pXF495-497: NMD3 promoter, NMD3 (TAG codon), NMD3 terminator

Note: The blue corresponding codon were individually replaced with the TAG stop codon via site-directed mutagenesis.

tcgcgcgtttcggtgatgacggtgaaaacctctgacacatgcagctcccggagacggtcacagcttgtctgtaagcggatgccgggagcagacaagcccgtcagg
gcgcgtcagcgggtgttggcgggtgtcggggctggcttaactatgcggcatcagagcagattgtactgagagtgcaccataccacagcttttcaattcaattcatcattttt
tttttattctttttttgatttcggtttctttgaaatttttttgattcggtaatctccgaacagaaggaagaacgaaggaaggagcacagacttagattggtatatatacgcatatgt
agtgttgaagaaacatgaaattgcccagtattcttaacccaactgcacagaacaaaaacctgcaggaaacgaagataaatcatgtcgaaagctacatataaggaa
cgtgctgctactcatcctagtcctgttgctgccaagctatttaatatcatgcacgaaaagcaaacaaacttgtgtgcttcattggatgttcgtaccaccaaggaattactgg
agttagttgaagcattaggtcccaaaatttgtttactaaaaacacatgtggatatcttgactgatttttccatggagggcacagttaagccgctaaaggcattatccgccaa
gtacaattttttactcttcgaagacagaaaatttgctgacattggtaatacagtcaaattgcagtactctgcgggtgtatacagaatagcagaatggcagacattacga
atgcacacggtgtggtgggcccaggtattgttagcggtttgaagcaggcggcagaagaagtaacaaaggaacctagaggccttttgatgttagcagaattgtcatgc
aagggctccctatctactggagaatatactaagggtactgttgacattgcgaagagcgacaaagattttgttatcggctttattgctcaaagagacatgggtggaagag
atgaaggttacgattggttgattatgacacccggtgtgggtttagatgacaagggagacgcattgggtcaacagtatagaaccgtggatgatgtggtctctacaggatct
gacattattattgttggaagaggactatttgcaaagggaagggatgctaaggtagagggtgaacgttacagaaaagcaggctgggaagcatatttgagaagatgcg
gccagcaaaactaaaaaactgtattataagtaaatgcatgtatactaaactcacaaattagagcttcaatttaattatatcagttattaccctatgcggtgtgaaataccgc
acagatgcgtaaggagaaaataccgcatcaggaaattgtaaacgttaatattttgttaaaattcgcgttaaatttttgttaaatcagctcattttttaaccaataggccgaaa
tcggcaaaatcccttataaatcaaaagaatagaccgagatagggttgagtgttgttccagtttggaacaagagtccactattaaagaacgtggactccaacgtcaaag
ggcgaaaaaccgtctatcagggcgatggcccactacgtgaaccatcaccctaatcaagttttttggggtcgaggtgccgtaaagcactaaatcggaaccctaaagg
gagcccccgatttagagcttgacggggaaagccggcgaacgtggcgagaaaggaagggaagaaagcgaaaggagcgggcgctagggcgctggcaagtgta
gcggtcacgctgcgcgtaaccaccacacccgccgcgcttaatgcgccgctacagggcgcgtcgcgccattcgccattcaggctgcgcaactgttgggaagggcga
tcggtgcgggcctcttcgctattacgccagctggcgaaagggggatgtgctgcaaggcgattaagttgggtaacgataccgctgtaataagaaaagccttgaccaag
gtaaccaaggcgaagagggaatcgctgatggcaatgcaaaaggaaaacttcggtgagagaaaaagacagcaaaagaagaagcaaaatgacgggaaaagtt
tgcgctcttgaaaaattctaaataagatttaaagacgtacatatcgtacaatctggatactgttttccaggacaactgatagatacaattttggcacatatgaagtatgtttct
atataacgggtgagaaaatcgctgtaaattcaattacgtacaatcaccgacgggtaacttatttttttttctttttctcttgtagttggaaaatttttttgaagctcatcgcattgga
aaagaaaaattactagcagttgtgatgatttaactatgttttgaggaaccttggctttatcttttatttgtcgcctggtacataacactacattttattgtctaataaacgttgagg
aatttgacaaaaggatggaattcacacctatagatccgcaccagcaccaaaatgctgctactcttctatgttgtaactgtggtaccccaatcgatgggtccactggctg
gttatgtgttatgactgtatcaaactgactgtggacattactcaaggtattccaagagaagcgaacatttcattttgcagaaactgtgaaagatttttgcaaccacctggac

aatggatcagagcagaattagaatctagagaattactggccatttgtttgcgtcgtttgaagggcctaacaaaagtcagattagtggatgcttcattcatttggacagaac
cccattctagacgtattagaattaaacttaccgtgcagggagaagccatgacaaatactattattcagcaaactttcgaagtagaatacattgttattgcgatgcaatgtc
cagactgtgccagatcctacactacaaatacttggagggctactgtccaaattagacaaaaagtgcctcacaagagaacatttttgtttttggaacaactgattttgaaa
cataatgctcatgtggataccatttccattagtgaagctaaggacgggttggatttcttctatgcgcaaaaaaaccacgcagttaagatgattgatttttgaacgctgttgt
ccgattaaacataaaaagtctgaagaacttatttctcaagatacacatactggtgcatctacttataaattttcatattctgtcgagattgttcctatctgtaaagatgacttggt
cgttttaccaaaaaaactggctaaatctatgggtaacatttctcaatttgttctatgttctaagatttctaacactgtccagtttatggatccgactactttgcaaaccgcggatc
tgtcaccatctgtgtactggagggctccattcaacgccctggctgatgtgacccagttggtggagttcattgttctggacgtggattctacaggtataagcaggggaaatc
gtgtcttagctgacattactgttgctagaacgtctgacttgggtgttaatgaccaagtctactacgtcaggtctcatctcggtggtatttgccatgcaggtgatagtgttatggg
ttatttcattgcaaactcgaattacaactcagatctttttgatgggttaaatatcgattatgtgcctgatgtagttcttgtgaagaaattgtatcaaagaaagagcaaaaaga
gcagacactggaagttaaagagaatggctaaggaacacaaagatatcgatgcatccttagattacaattcaagagctcaaaagcaagaaatggaacgtgcagaa
aaggactacgaattattcttacaagaattagaagaggatgctgaattaagacaaagtgttaacctatacaaaaaccgcgaagcaaatgtacctcctgaagagcatg
aaatggatgaagacgaggatgaagacgctccacaaatcaacattgatgaattattggacgagttagatgaaatgacattagaagatggagtcgagaacacacccg
ttgaatctcagcagtaaaaaaacaccaaagaatggagagtagtatatactcttaacaaacactagcatttcagtttatctatatacatatatatggatgtaaaatgtacttt
atggaagaacaagccaccacatgttgaaaactagataggcaagcaagatttttcatttgtagagctcttacgcaagatttttaaacttccgccgtaatcatggtcatagct
gtttcctgtgtgaaattgttatccgctcacaattccacacaacataggagccggaagcataaagtgtaaagcctggggtgcctaatgagtgaggtaactcacattaattg
cgttgcgctcactgcccgctttccagtcgggaaacctgtcgtgccagctgcattaatgaatcggccaacgcgcggggagaggcggtttgcgtattgggcgctcttccgc
ttcctcgctcactgactcgctgcgctcggtcgttcggctgcggcgagcggtatcagctcactcaaaggcggtaatacggttatccacagaatcaggggataacgcagg
aaagaacatgtgagcaaaaggccagcaaaaggccaggaaccgtaaaaaggccgcgttgctggcgtttttccataggctccgcccccctgacgagcatcacaaa
aatcgacgctcaagtcagaggtggcgaaacccgacaggactataaagataccaggcgtttccccctggaagctccctcgtgcgctctcctgttccgaccctgccgctt
accggatacctgtccgcctttctcccttcgggaagcgtggcgctttctcatagctcacgctgtaggtatctcagttcggtgtaggtcgttcgctccaagctgggctgtgtgca
cgaaccccccgttcagcccgaccgctgcgccttatccggtaactatcgtcttgagtccaacccggtaagacacgacttatcgccactggcagcagccactggtaaca
ggattagcagagcgaggtatgtaggcggtgctacagagttcttgaagtggtggcctaactacggctacactagaaggacagtatttggtatctgcgctctgctgaagcc
agttaccttcggaaaaagagttggtagctcttgatccggcaaacaaaccaccgctggtagcggtggtttttttgtttgcaagcagcagattacgcgcagaaaaaaagg
atctcaagaagatcctttgatcttttctacggggtctgacgctcagtggaacgaaaactcacgttaagggattttggtcatgagattatcaaaaaggatcttcacctagatc
cttttaaattaaaaatgaagttttaaatcaatctaaagtatatatgagtaaacttggtctgacagttaccaatgcttaatcagtgaggcacctatctcagcgatctgtctatttc
gttcatccatagttgcctgactccccgtcgtgtagataactacgatacgggagggcttaccatctggccccagtgctgcaatgataccgcgagacccacgctcaccgg
ctccagatttatcagcaataaaccagccagccggaagggccgagcgcagaagtggtcctgcaactttatccgcctccatccagtctattaattgttgccgggaagcta
gagtaagtagttcgccagttaatagtttgcgcaacgttgttgccattgctacaggcatcgtggtgtcacgctcgtcgtttggtatggcttcattcagctccggttcccaacgat
caaggcgagttacatgatcccccatgttgtgcaaaaaagcggttagctccttcggtcctccgatcgttgtcagaagtaagttggccgcagtgttatcactcatggttatgg
cagcactgcataattctcttactgtcatgccatccgtaagatgcttttctgtgactggtgagtactcaaccaagtcattctgagaatagtgtatgcggcgaccgagttgctctt
gcccggcgtcaatacgggataataccgcgccacatagcagaactttaaaagtgctcatcattggaaaacgttcttcggggcgaaaactctcaaggatcttaccgctgt
tgagatccagttcgatgtaacccactcgtgcacccaactgatcttcagcatcttttactttcaccagcgtttctgggtgagcaaaaacaggaaggcaaaatgccgcaaa
aaagggaataagggcgacacggaaatgttgaatactcatactcttcctttttcaatattattgaagcatttatcagggttattgtctcatgagcggatacatatttgaatgtatt
tagaaaaataaacaaataggggttccgcgcacatttccccgaaaagtgccacctgggtccttttcatcacgtgctataaaaataattataatttaaatttttaatataaata
tataaattaaaaatagaaagtaaaaaaagaaattaaagaaaaaatagtttttgttttccgaagatgtaaaagactctaggggggatcgccaacaaatactaccttttatct
tgctcttcctgctctcaggtattaatgccgaattgtttcatcttgtctgtgtagaagaccacacacgaaaatcctgtgattttacattttacttatcgttaatcgaatgtatatctatt
taatctgcttttcttgtctaataaatatatatgtaaagtacgctttttgttgaaattttttaaacctttgtttatttttttttcttcattccgtaactcttctaccttctttatttactttctaaaatc
caaatacaaaacataaaaataaataaacacagagtaaattcccaaattattccatcattaaaagatacgaggcgcgtgtaagttacaggcaagcgatccgtcctaa
gaaaccattattatcatgacattaacctataaaaataggcgtatcacgaggccctttcgtc

pXF469/pXF500: GAL1 promoter, SpCas9-NG, ADH1 terminator, SNR52 promoter, gRNA1, SUF17, gRNA2, SNR52 terminator, sup8

ggatggacgcaaagaagtttaataatcatattacatggcattaccaccatatacatatccatatctaatcttacttatatgttgtggaaatgtaaagagccccattatcttag
cctaaaaaaaccttctctttggaactttcagtaatacgcttaactgctcattgctatattgaagtacggattagaagccgccgagcgggcgacagccctccgacggaag
actctcctccgtgcgtcctcgtcttcaccggtcgcgttcctgaaacgcagatgtgcctcgcgccgcactgctccgaacaataaagattctacaatactagcttttatggttat
gaagaggaaaaattggcagtaacctggccccacaaaccttcaaattaacgaatcaaattaacaaccataggatgataatgcgattagttttttagccttatttctggggt
aattaatcagcgaagcgatgatttttgatctattaacagatatataaatggaaaagctgcataaccactttaactaatactttcaacattttcagtttgtattacttcttattcaa
atgtcataaaagtatcaacaaaaaattgttaatatacctctatactttaacgtcaaggagaaaaaactataatggattataaagatgacgatgacaaacctccaaaaa
agaagagaaaggtcgataagaaatactcaataggcttagatatcggcacaaatagcgtcggatgggcggtgatcactgatgaatataaggttccgtctaaaaagttc
aaggttctgggaaatacagaccgccacagtatcaaaaaaaatcttataggggctcttttatttgacagtggagagacagcggaagcgactcgtctcaaacggacag
ctcgtagaaggtatacacgtcggaagaatcgtatttgttatctacaggagatttttcaaatgagatggcgaaagtagatgatagtttctttcatcgacttgaagagtcttttt
ggtggaagaagacaagaagcatgaacgtcatcctatttttggaaatatagtagatgaagttgcttatcatgagaaatatccaactatctatcatctgcgaaaaaaattgg
tagattctactgataaagcggatttgcgcttaatctatttggccttagcgcatatgattaagtttcgtggtcatttttttgattgagggagatttaaatcctgataatagtgatgtgg
acaaactatttatccagttggtacaaacctacaatcaattatttgaagaaaaccctattaacgcaagtggagtagatgctaaagcgattctttctgcacgattgagtaaat
caagacgattagaaaatctcattgctcagctccccggtgagaagaaaaatggcttatttgggaatctcattgctttgtcattgggtttgacccctaattttaaatcaaattttg
atttggcagaagatgctaaattacagctttcaaaagatacttacgatgatgatttagataatttattggcgcaaattggagatcaatatgctgatttgtttttggcagctaaga
atttatcagatgctattttactttcagatatcctaagagtaaatactgaaataactaaggctcccctatcagcttcaatgattaaacgctacgatgaacatcatcaagacttg
actcttttaaaagctttagttcgacaacaacttccagaaaagtataaagaaatcttttttgatcaatcaaaaaacggatatgcaggttatattgatgggggagctagccaa
gaagaattttataaatttatcaaaccaattttagaaaaaatggatggtactgaggaattattggtgaaactaaatcgtgaagatttgctgcgcaagcaacggacctttga
caacggctctattccacatcaaattcacttgggtgagctgcatgctattttgagaagacaagaagacttttatccatttttaaaagacaatcgtgagaagattgaaaaaat
cttgacttttcgaattccttattatgttggtccattggcgcgtggcaatagtcgttttgcatggatgactcggaagtctgaagaaacaattaccccatggaattttgaagaagtt

gtcgataaaggtgcttcagctcaatcatttattgaacgcatgacaaactttgataaaaatcttccaaatgaaaaagtactaccaaaacatagtttgctttatgagtattttac
ggtttataacgaattgacaaaggtcaaatatgttactgaaggaatgcgaaaaccagcatttctttcaggtgaacagaagaaagccattgttgatttactcttcaaaacaa
atcgaaaagtaaccgttaagcaattaaaagaagattatttcaaaaaaatagaatgttttgatagtgttgaaatttcaggagttgaagatagatttaatgcttcattaggtac
ctaccatgatttgctaaaaattattaaagataaagatttttggataatgaagaaaatgaagatatcttagaggatattgttttaacattgaccttatttgaagatagggagat
gattgaggaaagacttaaaacatatgctcacctctttgatgataaggtgatgaaacagcttaaacgtcgccgttatactggttggggacgtttgtctcgaaaattgattaat
ggtattagggataagcaatctggcaaaacaatattagatttttgaaatcagatggttttgccaatcgcaattttatgcagctgatccatgatgatagtttgacatttaaaga
agacattcaaaaagcacaagtgtctggacaaggcgatagtttacatgaacatattgcaaatttagctggtagccctgctattaaaaaaggtattttacagactgtaaaa
gttgttgatgaattggtcaaagtaatggggcggcataagccagaaaatatcgttattgaaatggcacgtgaaaatcagacaactcaaaagggccagaaaaattcgc
gagagcgtatgaaacgaatcgaagaaggtatcaaagaattaggaagtcagattcttaaagagcatcctgttgaaaatactcaattgcaaaatgaaaagctctatctct
attatctccaaaatggaagagacatgtatgtggaccaagaattagatattaatcgtttaagtgattatgatgtcgatcacattgttccacaaagtttccttaaagacgattca
atagacaataaggtcttaacgcgttctgataaaaatcgtggtaaatcggataacgttccaagtgaagaagtagtcaaaaagatgaaaaactattggagacaacttcta
aacgccaagttaatcactcaacgtaagtttgataatttaacgaaagctgaacgtggaggtttgagtgaacttgataaagctggttttatcaaacgccaattggttgaaact
cgccaaatcactaagcatgtggcacaaattttggatagtcgcatgaatactaaatacgatgaaaatgataaacttattcgagaggttaaagtgattaccttaaaatctaa
attagtttctgacttccgaaaagatttccaattctataaagtacgtgagattaacaattaccatcatgcccatgatgcgtatctaaatgccgtcgttggaactgctttgattaa
gaaatatccaaaacttgaatcggagtttgtctatggtgattataaagtttatgatgttcgtaaaatgattgctaagtctgagcaagaaataggcaaagcaaccgcaaaat
atttcttttactctaatatcatgaacttcttcaaaacagaaattacacttgcaaatggagagattcgcaaacgccctctaatcgaaactaatggggaaactggagaaattg
tctgggataaagggcgagattttgccacagtgcgcaaagtattgtccatgccccaagtcaatattgtcaagaaaacagaagtacagacaggcggattctccaagga
gtcaattagaccaaaaagaaattcggacaagcttattgctcgtaaaaaagactgggatccaaaaaaatatggtggttttgttagtccaacggtagcttattcagtcctag
tggttgctaaggtggaaaaagggaaatcgaagaagttaaaatccgttaaagagttactagggatcacaattatggaaagaagttcctttgaaaaaaatccgattgact
ttttagaagctaaaggatataaggaagttaaaaaagacttaatcattaaactacctaaatatagtctttttgagttagaaaacggtcgtaaacggatgctggctagtgcc
agatttttacaaaaggaaatgagctggctctgccaagcaaatatgtgaatttttttatatttagctagtcattatgaaaagttgaagggtagtccagaagataacgaaca
aaaacaattgtttgtggagcagcataagcattatttagatgagattattgagcaaatcagtgaattttctaagcgtgttattttagcagatgccaatttagataaagttcttagt
gcatataacaaacatagagacaaaccaatacgtgaacaagcagaaaatattattcatttatttacgttgacgaatcttggagctcccagagcttttaaatattttgataca
acaattgatcgtaaagtttatagatctacaaaagaagttttagatgccactcttatccatcaatccatcactggtctttatgaaacacgcattgatttgagtcagctaggagg
tgaccctccaaaaaagaagagaaaggtctgactcgaggcgaatttcttatgatttatgatttttattattaaataagttataaaaaaaataagtgtatacaaattttaaagtg
actcttaggttttaaaacgaaaattcttattcttgagtaactctttcctgtaggtcaggttgctttctcaggtatagccacaccgcatagatccgtcgagttcaagagaaaaa
aaaagaaaaagcaaaaagaaaaaaggaaagcgcgcctcgttcagaatgacacgtatagaatgatgcattaccttgtcatcttcagtatcatactgttcgtatacatac
ttactgacattcataggtatacatatatacacatgtatatatatcgtatgctgcagctttaaataatcggtgtcactacataagaacacctttggtggagggaacatcgttgg
taccattgggcgaggtggcttctcttatggcaaccgcaagagccttgaacgcactctcactacggtgatgatcattcttgcctcgcagacaatcaacgtggagggtaatt
ctgctagcctctgcaaagctttcaagaaaatgcgggatcatctcgcaagagagatctcctactttctccctttgcaaaccaagttcgacaactgcgtacggcctgttcga
aagatctaccaccgctctggaaagtgcctcatccaaaggcgcaaatcctgatccaaacctttttactccacgcgccagtagggcctctttaaaagcttgaccgagagc
aatcccgcagtcttcagtggtgtgatggtcgtctatgtgtaagtcaccaatgcactcaacgattagcgaccagccggaatgcttggccagagcatgtatcatatggtcca
gaaaccctatacctgtgtggacgttaatcacttgcgattgtgtggcctgttctgctactgcttctgcctctttttctgggaagatcgagtgctctatcgctaggggaccaccctttt
aaagagatcgcaatctgaatcttggtttcatttgtaatacgctttactagggctttctgctctgtcatctttgccttcgtttatcttgcctgctcatttttagtatattcttcgaagaaa
tcacattactttatataatgtataattcattatgtgataatgccaatcgctaagaaaaaaaaagagtcatccgctaggggaaaaaaaaaaatgaaaatcattaccgagg
acagcttgtctgtaagcggatgccgggagcagacaagcccgtcagggcgcgtcagcgggtgttggcgggtgtcggggctggcgggaaaggactgtgttatgacttc
cctgactaatgcgatccaatatcaaaggaaatgatagcattgaaggatgagactaatccaattgaggagtggcagcatatagaacagctaaagggtagtgctgaag
gaagcatacgataccccgcatggaatgggataatatcacaggaggtactagactacctttcatcctacataaatagacgcatataagtacgcatttaagcataaaca
cgcactatgccgttcttctcatgtatatatatatacaggcaacacgcagatataggtgcgacgtgaacagtgagctgtatgtgcgcagctcgcgttgcattttcggaagcg
ctcgttttcggaaacgctttgaagttcctattccgaagttcctattctctagaaagtataggaacttcagagcgcttttgaaaaccaaaagcgctctgaagacgcactttca
aaaaaccaaaaacgcaccggactgtaacgagctactaaaatattgcgaataccgcttccacaaacattgctcaaaagtatctctttgctatatatctctgtgctatatcc
ctatataacctacccatccacctttcgctccttgaacttgcatctaaactcgacctctacattttttatgtttatctctagtattactctttagacaaaaaaaattgtagtaagaact
attcatagagtgaatcgaaaacaatacgaaaatgtaaacatttcctatacgtagtatatagagacaaaatagaagaaaccgttcataattttctgaccaatgaagaatc
atcaacgctatcactttctgttcacaaagtatgcgcaatccacatcggtatagaatataatcggggatgcctttatcttgaaaaaatgcacccgcagcttcgctagtaatc
agtaaacgcgggaagtggagtcaggctttttttatggaagagaaaatagacaccaaagtagccttcttctaaccttaacggacctacagtgcaaaaagttatcaaga
gactgcattatagagcgcacaaaggagaaaaaaagtaatctaagatgctttgttagaaaaatagcgctctcgggatgcatttttgtagaacaaaaaagaagtataga
ttctttgttggtaaaatagcgctctcgcgttgcatttctgttctgtaaaaatgcagctcagattctttgtttgaaaaattagcgctctcgcgttgcatttttgttttacaaaaatgaag
cacagattcttcgttggtaaaatagcgctttcgcgttgcatttctgttctgtaaaaatgcagctcagattctttgtttgaaaaattagcgctctcgcgttgcatttttgttctacaaa
atgaagcacagatgcttcgttagatctggccaggaaccgtaaaaaggccgcgttgctggcgtttttccataggctccgcccccctgacgagcatcacaaaaatcgac
gctcaagtcagaggtggcgaaacccgacaggactataaagataccaggcgtttccccctggaagctccctcgtgcgctctcctgttccgaccctgccgcttaccggat
acctgtccgcctttctcccttcgggaagcgtggcgctttctcatagctcacgctgtaggtatctcagttcggtgtaggtcgttcgctccaagctgggctgtgtgcacgaaccc
cccgttcagcccgaccgctgcgccttatccggtaactatcgtcttgagtccaacccggtaagacacgacttatcgccactggcagcagccactggtaacaggattagc
agagcgaggtatgtaggcggtgctacagagttcttgaagtggtggcctaactacggctacactagaagaacagtatttggtatctgcgctctgctgaagccagttacctt
cggaaaaagagttggtagctcttgatccggcaaacaaaccaccgctggtagcggtggtttttttgtttgcaagcagcagattacgcgcagaaaaaaaggatctcaag
aagatcctttgatcttttctacggggtctgacgctcagtggaacgaaaactcacgttaagggattttggtcatgagattatcaaaaaggatcttcacctagatccttttaaatt
aaaaatgaagttttaaatcaatctaaagtatatatgagtaaacttggtctgacagttaccaatgcttaatcagtgaggcacctatctcagcgatctgtctatttcgttcatcca
tagttgcctgactccccgtcgtgtagataactacgatacgggagggcttaccatctggccccagtgctgcaatgataccgcggctcccacgctcaccggctccagattt
atcagcaataaaccagccagccggaagggccgagcgcagaagtggtcctgcaactttatccgcctccatccagtctattaattgttgccgggaagctagagtaagta
gttcgccagttaatagtttgcgcaacgttgttgccattgctacaggcatcgtggtgtcacgctcgtcgtttggtatggcttcattcagctccggttcccaacgatcaaggcga
gttacatgatcccccatgttgtgcaaaaaagcggttagctccttcggtcctccgatcgttgtcagaagtaagttggccgcagtgttatcactcatggttatggcagcactgc
ataattctcttactgtcatgccatccgtaagatgcttttctgtgactggtgagtactcaaccaagtcattctgagaatagtgtatgcggcgaccgagttgctcttgcccggcgt
caatacgggataataccgcgccacatagcagaactttaaaagtgctcatcattggaaaacgttcttcggggcgaaaactctcaaggatcttaccgctgttgagatcca

gttcgatgtaacccactcgtgcacccaactgatcttcagcatcttttactttcaccagcgtttctgggtgagcaaaaacaggaaggcaaaatgccgcaaaaaagggaa
taagggcgacacggaaatgttgaatactcatactcttcctttttcaatattattgaagcatttatcagggttattgtctcatgagcggatacatatttgaatgtatttagaaaaat
aaacaaataggggttccgcgcacatttccccgaaaagtgccacctgaacggtcgacgcggccgcacaatctttgaaaagataatgtatgattatgctttcactcatattt
atacagaaacttgatgttttctttcgagtatatacaaggtgattacatgtacgtttgaagtacaactctagattttgtagtgccctcttgggctagcggtaaaggtgcgcattttt
tcacaccctacaatgttctgttcaaaagattttggtcaaacgctgtagaagtgaaagttggtgcgcatgtttcggcgttcgaaacttctccgcagtgaaagataaatgatc
gatctcaagatttcgtagtggtaaattgttttagagctagaaatagcaagttaaaataaggctagtccgttatcaacttgaaaaagtggcaccgagtcggtgcaaacaa
gcgcaagtggtttagtggtaaaatccaacgttgccatcgttgggcccccggttcgattccgggcttgcgcagatctgagaccggtctcagatctcaagatttcgtagtgat
aaattgttttagagctagaaatagcaagttaaaataaggctagtccgttatcaacttgaaaaagtggcaccgagtcggtgcttttttttatttttgtcactattgttatgtaaaat
gccacctctgacagtatggaacgcaaacttctgtctagtggataacagaatttttctatggccaatttactctcggtagccaagttggtttaaggcgcaagactgtaattta
ccactacgaaatcttgagatcgggcgttcgactcgcccccgggagagagagtgcaccataccacagcttttc

Supplemental References

1. Stieglitz, J.T., Potts, K.A. & Van Deventer, J.A. Broadening the Toolkit for Quantitatively Evaluating Noncanonical Amino Acid Incorporation in Yeast. *ACS Synth Biol* **10**, 3094-3104 (2021).
2. Li, Z. et al. Systematic exploration of essential yeast gene function with temperature-sensitive mutants. *Nat Biotechnol* **29**, 361-7 (2011).
