# Supplementary material for: A robust yeast biocontainment system with two-layered regulation switch dependent on unnatural amino acid
Source: Nat Commun. 2023 Oct 14;14:6487. doi: 10.1038/s41467-023-42358-4 (PMC10576815; doi:10.1038/s41467-023-42358-4)
Supplement: Supplementary file 2 — Reporting Summary [file 41467_2023_42358_MOESM2_ESM.pdf]

nature portfolio

Corresponding author(s): Xian Fu and Yue Shen

Last updated by author(s): Oct 8, 2023

Reporting Summary

Nature Portfolio wishes to improve the reproducibility of the work that we publish. This form provides structure for consistency and transparency in reporting. For further information on Nature Portfolio policies, see our Editorial Policies and the Editorial Policy Checklist.

Statistics

For all statistical analyses, confirm that the following items are present in the figure legend, table legend, main text, or Methods section.

n/a	Confirmed	
☐	☒	The exact sample size (*n*) for each experimental group/condition, given as a discrete number and unit of measurement
☐	☒	A statement on whether measurements were taken from distinct samples or whether the same sample was measured repeatedly
☐	☒	The statistical test(s) used AND whether they are one- or two-sided<br>*Only common tests should be described solely by name; describe more complex techniques in the Methods section.*
☒	☐	A description of all covariates tested
☒	☐	A description of any assumptions or corrections, such as tests of normality and adjustment for multiple comparisons
☐	☒	A full description of the statistical parameters including central tendency (e.g. means) or other basic estimates (e.g. regression coefficient) AND variation (e.g. standard deviation) or associated estimates of uncertainty (e.g. confidence intervals)
☐	☒	For null hypothesis testing, the test statistic (e.g. *F*, *t*, *r*) with confidence intervals, effect sizes, degrees of freedom and *P* value noted<br>*Give P values as exact values whenever suitable.*
☒	☐	For Bayesian analysis, information on the choice of priors and Markov chain Monte Carlo settings
☒	☐	For hierarchical and complex designs, identification of the appropriate level for tests and full reporting of outcomes
☒	☐	Estimates of effect sizes (e.g. Cohen's *d*, Pearson's *r*), indicating how they were calculated

Our web collection on statistics for biologists contains articles on many of the points above.

Software and code

Policy information about availability of computer code

Data collection	Bioscreen C system was used to collect the doubling time data.<br>The essential protein sequences were obtained from the database created by Guri Giaever et al (https://doi.org/10.1038/nature00935).
Data analysis	Microsoft excel (v16.73) was used to analyze the doubling time,escape frequency,and relevant statistical calculation.<br>Snapgene (v6.0.2) was used for gene sequence analysis and primer design.<br>Whole genome sequencing data, the raw data was filtered by SOAPnuke (v2.1.1).The raw sequenced reads were then mapped against the reference sequence of BY4741 using BWA (v2.2.5).The genetic mutations containing SNPs, insertions, and deletions in the genome of the escapers were identified by GATK (v2.7) with default settings.<br>The essential protein sequences were aligned using a new generation of protein database search programs according to a published paper (https://doi.org/10.1093/nar/25.17.3389).

For manuscripts utilizing custom algorithms or software that are central to the research but not yet described in published literature, software must be made available to editors and reviewers. We strongly encourage code deposition in a community repository (e.g. GitHub). See the Nature Portfolio guidelines for submitting code & software for further information.

Data

Policy information about availability of data

All manuscripts must include a data availability statement. This statement should provide the following information, where applicable:

- Accession codes, unique identifiers, or web links for publicly available datasets
- A description of any restrictions on data availability
- For clinical datasets or third party data, please ensure that the statement adheres to our policy

The DNA sequencing data of this study has been deposited in the NCBI database under accession code PRJNA967796 (https://www.ncbi.nlm.nih.gov/search/all/?term=PRJNA967796) and CNSA (CNGB Nucleotide Sequence Archive) under accession number CNP0004223 (https://db.cngb.org/search/project/CNP0004223/). The conservation analysis for all essential proteins of S. cerevisiae compared against their specific orthologs by BLAST uses the NCBI built-in cluster-nr database (https://blast.ncbi.nlm.nih.gov/Blast.cgi?PROGRAM=blastp&PAGE_TYPE=BlastSearch). All data supporting the findings of this study are available within the manuscript file and its Supplementary Information files. Source data are provided with this paper.

Research involving human participants, their data, or biological material

Policy information about studies with human participants or human data. See also policy information about sex, gender (identity/presentation), and sexual orientation and race, ethnicity and racism.

Reporting on sex and gender	N/A
Reporting on race, ethnicity, or other socially relevant groupings	N/A
Population characteristics	N/A
Recruitment	N/A
Ethics oversight	N/A

Note that full information on the approval of the study protocol must also be provided in the manuscript.

Field-specific reporting

Please select the one below that is the best fit for your research. If you are not sure, read the appropriate sections before making your selection.

[x] Life sciences [ ] Behavioural & social sciences [ ] Ecological, evolutionary & environmental sciences

For a reference copy of the document with all sections, see nature.com/documents/nr-reporting-summary-flat.pdf

Life sciences study design

All studies must disclose on these points even when the disclosure is negative.

Sample size	For doubling time measurement, 3-6 biological replicates were performed for each strain as this sample size is standard for yeast growth assay (PMID: 28174266, 29789541). For each replicate in escape assay, three biological replicates were performed for each strain, and each of the three biological replicates was conducted in duplicate (two technical repetitions). These sample sizes are sufficient based on literatures about yeast-based biocontainment system (PMID: 33122649). Approximately 1 x 10(-9) escapees per CFUs was chosen as the detection limit based on the consideration of reasonable amount of works. Our assay limit reaches the high standard set according to literatures about yeast-based biocontainment system (PMID: 33122649 and 28174266). For fitness-oriented screening at N-terminus assay, 124 and 146 CDC4- and CDC27-based OMeY auxotrophs respectively were randomly collected for Sanger sequencing to determine TAG substitution site. For the potential escape mechanism, 18 independent escapers were selected randomly for whole genome sequencing (WGS) analysis. For fluorescence measurement, eight biological replicates were performed for each strain.
Data exclusions	No data were excluded in our study.
Replication	All escape assays and doubling time measurements were repeated 3-6 times independently as indicated in the figure legends and showed similar results.
Randomization	Clones with different colony size and growing in different areas on agar plate were all randomly picked up to avoid position effects and phenotypic effects. Replicates were randomized across micro-plate for growth measurement to avoid position effects.
Blinding	Blinding is not relevant because no group allocation was involved in this study.

Reporting for specific materials, systems and methods

We require information from authors about some types of materials, experimental systems and methods used in many studies. Here, indicate whether each material, system or method listed is relevant to your study. If you are not sure if a list item applies to your research, read the appropriate section before selecting a response.

Materials & experimental systems

n/a	Involved in the study
☒	☐ Antibodies
☒	☐ Eukaryotic cell lines
☒	☐ Palaeontology and archaeology
☒	☐ Animals and other organisms
☒	☐ Clinical data
☒	☐ Dual use research of concern
☒	☐ Plants

Methods

n/a	Involved in the study
☒	☐ ChIP-seq
☒	☐ Flow cytometry
☒	☐ MRI-based neuroimaging
